# Structural Model of the mIgM B-Cell Receptor Transmembrane Domain From Self-Association Molecular Dynamics Simulations

**DOI:** 10.3389/fimmu.2018.02947

**Published:** 2018-12-17

**Authors:** Mario D. Friess, Kristyna Pluhackova, Rainer A. Böckmann

**Affiliations:** Department of Biology, Computational Biology, Friedrich-Alexander University of Erlangen-Nürnberg, Erlangen, Germany

**Keywords:** B-cell receptor, transmembrane domain, nanodomains, self-assembly, molecular dynamics simulations, coarse-grained simulations, dissociation activation model

## Abstract

Antigen binding to B-cell antigen receptors (BCRs) followed by signaling initiates the humoral immune response. The signaling is intimately coupled to nanoclustering of BCRs and their sorting to specific membrane domains, a process that is ruled by interactions between the BCR transmembrane domain and lipids. While the structure of the extracellular domains of BCRs has been resolved, little is known about the configuration of the constituting four immunoglobulin domains spanning the membrane. Here, we modeled the structure of the transmembrane (TM) domain of the IgM B-cell receptor using self-assembly coarse-grained molecular dynamics simulations. The obtained quaternary structure was validated against available experimental data and atomistic simulations. The IgM-BCR-TM domain configuration shows a 1:1 stoichiometry between the homodimeric membrane-bound domain of IgM (mIgM) and a Ig-α/Ig-β heterodimer. The mIgM homodimer is based on an asymmetric association of two mIgM domains. We show that a specific site of the Ig-α/Ig-β heterodimer is responsible for the association of IgM-BCRs with lipid rafts. Our results further suggest that this site is blocked in small-sized IgM-BCR clusters. The BCR TM structure provides a molecular basis for the previously suggested dissociation activation model of B-cell receptors. Self-assembly molecular dynamics simulations at the coarse-grained scale here proved as a versatile tool in the study of receptor complexes.

## 1. Introduction

As one of the main parts of the adaptive immune sytem, B cells play a key role in the protection against pathogens. Defects during B-cell development and selection may lead to resistance against healthy tissue resulting in autoimmunity, malignancy, or allergy (1). B cells recognize and fight pathogens by the help of proteins called immunoglobulines (Ig). The five immunoglobuline isotypes (IgA, IgD, IgE, IgG, and IgM) can either be secreted (sIgs) or membrane-bound (mIgs) on the cell surface. The membrane-bound immunoglobulines (mIgA, mIgD, mIgE, mIgG, and mIgM) are components of the so-called B-cell receptors (BCR).

**Figure 1:**
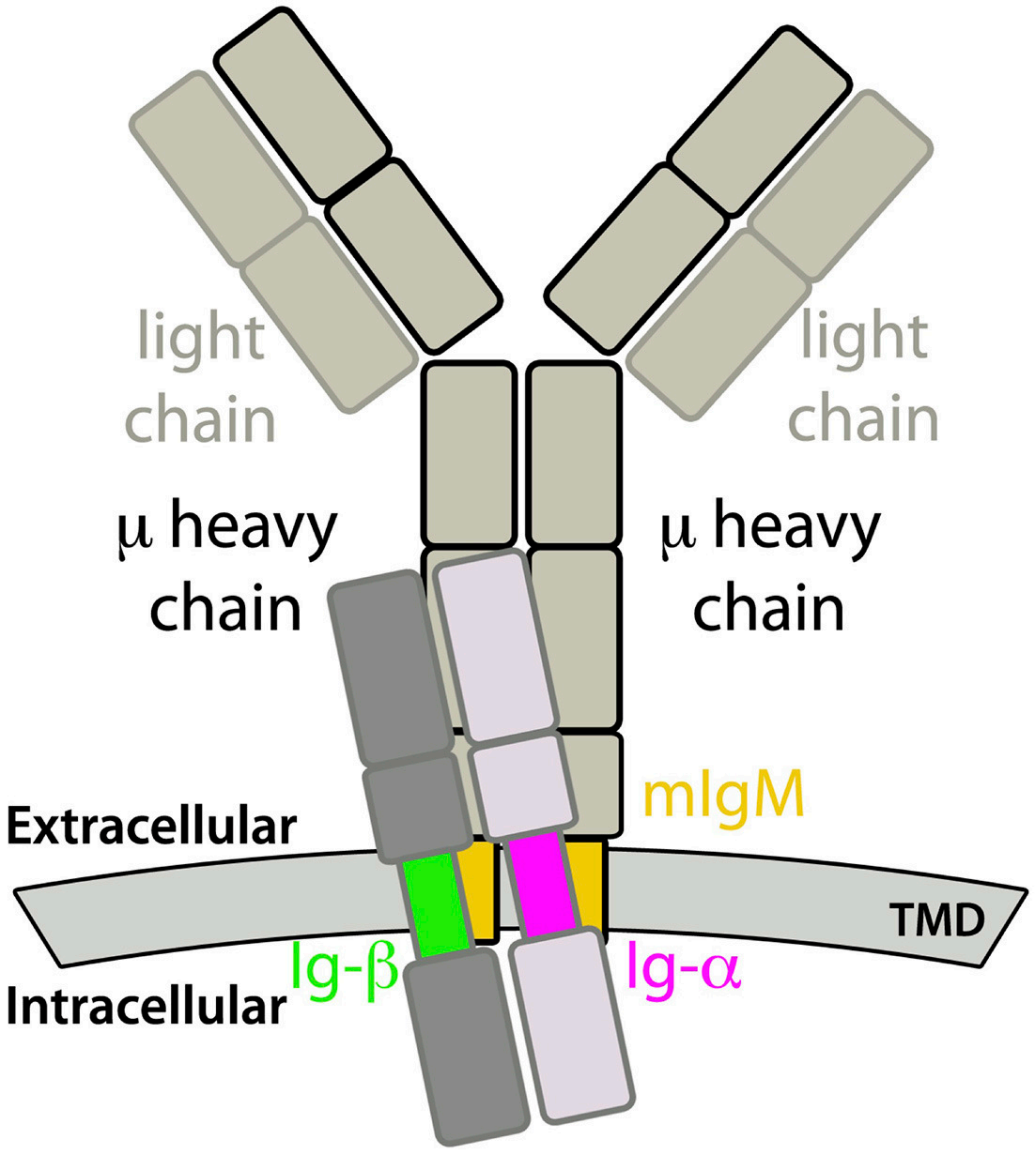
Sketch of a IgM-B cell receptor (BCR) complex. The transmembrane domains of mIgM and the Ig-α/Ig-β heterodimer are colored.

The membrane-anchorage of mIgs, which are tetramers consisting of two identical heavy (μ) and two identical light chains, is granted only by the C-terminal ends of both heavy chains. In case of mIgM, the C-terminal parts can be further divided into three domains, namely the extracellular membrane-proximal domain, followed by a transmembrane domain (TMD) and a cytoplasmic domain (2, 3). The task of mIgM is to respond to antigen binding by signal transmission across the plasma membrane leading to B-cell activation and consequently clonal expansion and specific antibody production. To that end, mIgMs non-covalently associate with the membrane-spanning Ig-α/Ig-β heterodimer, forming the fully functional IgM-BCR complex [see Figure 1; 4]. Thereby, the Ig-α/Ig-β-TMD and the mIgM-TMD specifically bind to each other (5). Ig-α as well as Ig-β contain a conserved immunoreceptor tyrosine-based activation motif (ITAM). These well-known signaling motifs are patterns of four amino acids in which a tyrosine is separated from a leucine or an isoleucine by any two residues (Y X X L/I). These motifs located in the cytoplasmic domain are generally repeated twice and separated by 7-12 residues (Y X X L/I 7-12 Y X X L/I) (6). Biochemical studies revealed a 1:1 stoichiometry between mIg and Ig-α/Ig-β (7), which was confirmed by fluorescence spectroscopy (8).

The assembly of the mIgM-TMD with Ig-α/Ig-β-TMD was shown to be crucial for surface expression and overcoming of endoplasmatic reticulum retention (5, 9). Later, the association was shown to be mediated by the YS motif (Y463, S464) inside the mIgM-TMD. Since mutation of Tyr463 of the YS motif to phenylalanine did not result in a detectable effect on association, rather the shape than the hydrophobic character mediated by the hydroxy groups is important for mIgM-TMD – Ig-α/Ig-β-TMD association (10–15).

So far there is no structural information available about the molecular architecture of the transmembrane domain of IgM-BCR (16). Nevertheless, the BCR-TMD is generally assumed to adopt an α-helical conformation (17). The α-helical scheme of the μ heavy chain TMD reveals two distinct sites: Amino acids at one site of the helix are conserved (TM-C site) between different mIg classes, while the other site of the helix is specific for the mIg-type (TM-S site) (17). Since all mIgs bind the same Ig-α/Ig-β heterodimer, this observation suggests that binding of Ig-α/Ig-β involves the conserved site of the mIgM TM helix, while mIg dimerization and class-specific BCR oligomerization (7) involve the specific part of the helix (17). A hypothetical symmetric homodimer between two μ heavy chains that dimerize via their TM-S sites, however, would leave the opposing, distal TM-C sites of mIgM uncovered and would therefore likely enable binding of two Ig-α/Ig-β domains and thus violate the observed 1:1 stoichiometry between mIgM and Ig-α/Ig-β. Additionally, this configuration would not account for the class-specific behavior of BCRs inside membranes (see below), which suggests that at least one specific binding site remains lipid-accessible after IgM-BCR assembly. This is in line with Yang and Reths observation, that mutations within the TM-S site strongly affect the ability of IgD-BCRs to build oligomers (18). Theoretical considerations would hence suggest an asymmetric binding mode, leaving one TM-C site uncovered for binding of the Ig-α/Ig-β heterodimer in a 1:1 stoichiometry and one uncovered TM-S region for class-specific behavior and/or oligomerization process (see below).

Several studies and models couple the activation mechanism of BCRs and their supraorganisation on the cell surface: The cross-linking model states that cross-linking of separated, inactive receptors, e.g., with the assistance of a bivalent antigen, leads to receptor activation. This model could well explain the discovery that only bivalent but not monovalent antigen binding fragments are able to trigger BCR-signaling (18–23). However, Yang and Reth (18) pointed out several conflicts between the cross-linking model and BCR properties and proposed the dissociation activation model (DAM), in which auto-inhibited BCR-oligomers are shifted into the active state via antigen binding and subsequent cluster opening, leading to freely accessible ITAM motifs and exposure of the TM region to the lipid environment (6, 24). The ITAM motifs would then be accessible for kinases like Syk or Lyn, while the TM region would be in contact with the lipid environment.

The membrane composition was suggested to play a key role in BCR activation. Both IgD- and IgM-BCRs were reported to form oligomers or nanoclusters of different sizes (7, 25). However, BCRs display a class-specific and activation-dependent membrane (nano-)domain preference: Activated IgM-BCRs as well as resting IgD-BCRs were found to reside in ganglioside-enriched membrane domains, whereas resting IgM-BCRs and activated IgD-BCRs were not. Thus, BCR activation is accompanied by a modulation of the lipid nano-enrivonment of the BCRs (26). Since BCRs show a class-specific preference for the lipid composition, and related to the finding that protein-lipid interactions drive the localization of TM proteins in membranes (27), the TM-S region of mIg likely participates in BCR localization, i.e., at least one TM-S site should interact with the lipids instead of taking part in mIg:Ig-α/Ig-β-assembly.

The clustering of resting BCRs, as stated by the DAM, might be explained by clustering of polar amino acids in order to shield them from the hydrophobic lipid environment (28). Without any antagonistic force, evasion from the energetically unfavorable monomeric state and aggregation of hydrophilic areas is a plausible scenario (28, 29). The burial of polar TMD-areas could be controlled and stabilized by changes in the lipid environment, taking part in the BCR cluster formation and cluster opening processes. Alternatively, specific protein—lipid interactions may as well stabilize the BCR monomeric state and prevent reassembly, as it is required for BCR activation. This is in line with a recent *in silico* study of the dimerization of the G protein coupled receptor CXCR4, which revealed a cholesterol-dependent dimerization site that could be blocked by cholesterol (30).

Motivated by the reported major role of the TMD in mIgM – Ig-α/Ig-β assembly as well as in IgM-BCR oligomerization and the association of resting or active BCRs with different membrane nanodomains, this study focuses on the quaternary configuration of the IgM-BCR-TM domain and the underlying mechanisms of IgM-BCR-TMD – lipid raft association upon BCR activation. Ensembles of coarse-grained molecular dynamics (MD) simulations were employed to study the spontaneous association of the TM domains of mIgM and of Ig-α/Ig-β, accompanied by atomistic-scale MD simulations addressing the stability of obtained quarternary structures. This approach was shown before to yield excellent results for the dimerization and oligomerization of TM peptides (31–33) but as well for the homo- and heterodimerization of GPCRs (30, 34), or the adsorption of peptides to membrane interfaces (35).

We report a BCR-TMD configuration that is in agreement with the available experimental data. The Ig-α/Ig-β lipid interface is shown to drive the association of IgM-BCR-TMD to lipid raft-like domains. Shielding of this interface upon IgM-BCR oligomerization is suggested to suspend BCR lipid raft association; In turn, BCR cluster opening upon activation would shift the preferred BCR-TMD environment toward the disordered membrane phase.

## 2. Materials and Methods

### 2.1. Coarse-Grained Simulations

The transmembrane domain of the B-cell receptor consists of four TM α-helices: the mIgM TMD homodimer and one α-helix of Ig-α and Ig-β, respectively. Association of the BCR-TMD was addressed in separate self-assembly MD simulations of the mIgM TMD monomers to a homodimer, of the Ig-α and Ig-β helices to a heterodimer, and finally of the pre-assembled mIgM TMD homodimer and the Ig-α/Ig-β heterodimer to the full IgM-BCR-TMD (see also Figure 1).

All coarse-grained simulations were prepared using the docking assay for transmembrane components (DAFT) scheme (31), combined with the Gromacs 4.6 simulation suite (36) and the coarse-grained MARTINI force field (37, 38). DAFT allows to efficiently setup a large number of oligomerization simulations starting from unbiased initial states. Thereby, ensembles of associating or non-associating proteins are obtained that mirror the underlying energy landscape and provide a converged view on protein-protein and protein-lipid interaction interfaces.

Input structures for self-association simulations of isolated TM α-helices were based on the sequences of the individual IgM-BCR components (Table 1). PyMOL (39) was used for modeling of the α-helical input structures (mIgM TMD, Ig-α and Ig-β TMDs; Table 2, Steps 1 and 2). Association of the full BCR TM complex was based on association simulations of pre-formed mIgM homodimer and the Ig-α/Ig-β heterodimer complexes (compare Results section, Table 2, Steps 3a, 3b, 5, and 6). Due to the high amount of charged residues surrounding the TMDs of Ig-α and Ig-β, all coarse-grained MD simulations containing Ig-α, Ig-β or an Ig-α/Ig-β heterodimer were carried out using the polarizable water model (40) (Table 2, Steps 2, 3a, 3b, 5, and 6) and the polarizable MARTINI protein force-field (41).

**Table 1:** Amino acid sequences of the transmembrane domains of mIgM, Ig-α, and Ig-β studied in coarse-grained and atomistic MD simulations.

**Molecule**	**Sequence**
mIgM	442-GFENLWATASTFIVLFLLSLFYSTTVTLFKVK-473
Ig-α	135-DMGEGTKNRIITAEGIILLFCAVVPGTLLLFRKRWQ-170
Ig-β	151-LKQRNTLKDGIIMIQTLLIILFIIVPIFLLLDKDDS-186

**Table 2:** List of coarse-grained (CG) and all-atom (AA) MD simulations performed to study the conformation of the IgM-BCR transmembrane domain.

**Step^**a**^**	**Aim**	**Resolution^**b**^**	**Simulation time^**c**^**	**Number of (Simulations)^**d**^**
1	mIgM homodimer assembly (resulting in BM-A and BM-B configurations)	CG	5000 ns	104
2	Ig-α/Ig-β heterodimer assembly (resulting in BM-α configuration)	CG	5000 ns	105
3a	IgM-BCR assembly of binding modes BM-A and BM-α	CG	5000 ns	176
3b	IgM-BCR assembly of binding modes BM-B and BM-α	CG	5000 ns	190
4	Stability assessment of BM-A, BM-B, BM-α, BM-A-1, BM-A-2, BM-B-1 and BM-B-2	AA	500 ns	7x3^e^
5	Membrane domain preference of different IgM-BCR TM configurations (BM-A-1, BM-A-2, BM-B-1, and BM-B-2)	CG	2000 ns	4x10^f^
6	Dimerization of IgM-BCR TM domains	CG	10000 ns	110
7	Stability assessment of IgM-BCR cluster	AA	500 ns	3

a *For clarity, the workflow was divided into 7 parts*.

b *Simulations were carried out either at all-atom (AA) or at coarse-grained (CG) resolution*.

c *Simulation time of each simulation*.

d *Number of replica simulations*.

e *Three all-atom simulations were performed for each IgM-BCR TM configuration (BM-A, BM-B, BM-α, BM-A-1, BM-A-2, BM-B-1, and BM-B-2)*.

f *10 CG simulations were performed for each IgM-BCR TM configuration (BM-A-1, BM-A-2, BM-B-1 and BM-B-2)*.

In simulations targeting the spontaneous self-assembly of two transmembrane domains, the TM helices/domains were embedded at a center of mass distance of 5 nm and random in-plane rotations in a 1-palmitoyl-2-oleoyl-sn-glycero-3-phosphocholine (POPC) membrane (Table 2, Steps 1, 2, 3a, 3b and 6). The membrane domain preference of the BCR was addressed for different BCR TMD models in simulations of the receptor embedded in 1,2-dipalmitoyl-sn-glycero-3-phosphocholine (DPPC)/1,2-di-(cis-cis-cis-9,12,15-octadecadienoyl)-sn-glycero-3-phosphocholine (DIPC)/cholesterol (proportions: 40:30:30) membranes as a model system for ordered/disordered membrane domains (Table 2, Step 4). The different lipids were initially randomly distributed within the membrane.

All systems were equilibrated according to the *MARTINATE* protocol (42). All production runs were then carried out in an (approximate) NpT ensemble with a timestep of 20 fs. The temperature was controlled by coupling to an external heat bath of 310 K with the aid of the Bussi velocity rescaling thermostat (43) and a coupling time constant of 1.0 ps. The Berendsen barostat (44) was used for semi-isotropic pressure coupling to an external pressure bath at 1 bar with a 3.0 ps coupling time constant and a compressibility of 3.0·10^−4^ bar^−1^. Lennard-Jones interactions were switched to zero between 0.9 and 1.2 nm. Bonds were constrained using LINCS (45).

In case of the non-polarizable MARTINI water model (Table 2, Step 1), the relative dielectric permittivity was set to 15 and electrostatic interactions were switched to zero between 0.0 and 1.2 nm. In contrast, in the case of the polarizable MARTINI water model (37) (Table 2, Steps 2, 3a, 3b, 5, and 6), a cut-off of 0.9 nm was applied for short-range electrostatic interactions and the PME method (46) was used for long-range electrostatics beyond the cutoff. Here, the relative dielectric permittivity was set to 2.5.

### 2.2. All-Atom Simulations

Atomistic simulations of BCR transmembrane domains were performed inside a 1-palmitoyl-2-oleoyl-sn-glycero-3-phosphoethanolamine (POPE) membrane, in order to assure for a comparable membrane thickness between models at coarse-grained (CG) and atomistic resolution (Table 2, Steps 4 and 7; see Results section). The *insane* protocol (47, 48) was used to setup the lipid and solvent environment around input structures at CG resolution. Equilibration at CG resolution employing the *DAFT* scheme (31) was followed by conversion of the whole system to atomistic resolution employing the *backward* protocol (49). For all systems, the minimal distance between periodic images of the proteins never decreased below 3 nm.

Atomistic simulation production runs of 500 ns length, three replicas for each system, using Gromacs 5 (50) were preceded by an energy minimization using the steepest descent algorithm. The systems were simulated in the NpT ensemble for 10 ns with restraints on all heavy protein atoms, and additionally for 5 ns with restraints on the protein backbone atoms only. A combination of the AMBER14sb force field (51) for proteins and the LIPID14 (52) force field for lipids was chosen (53, 54). Water was described by the TIP3P water model (55) and ions were added at physiological concentration (150 mM Na^+^Cl^−^).

The temperature was controlled by coupling to an external heat bath at 310 K using the Bussi velocity rescaling thermostat (43) and a coupling time constant of 0.5 ps. A pressure of 1 bar was reached by semi-isotropic pressure coupling to an external pressure bath [Berendsen barostat (44)] with a time constant of 1 ps. The compressibility was set to 4.5·10^−5^ bar^−1^. Lennard-Jones interactions and short-range electrostatic interactions were taken into account until a cut-off of 1 nm, while the PME method was used for long-range electrostatics beyond the cutoff. The production runs were carried out with a timestep of 2 fs. Bonds to hydrogen atoms were constrained by LINCS (45).

### 2.3. Analysis

As a dimerization criterium both for α-helices (mIgM, Ig-α, and Ig-β) and for helical dimers in the formation of the full BCR TMD (mIgM TMD homodimer and the Ig-α/Ig-β heterodimer) the interaction energy (sum of Lennard-Jones and Coulomb interactions) between two monomers was set to −200 kJ/mol. This cutoff value was chosen from visual inspection of the compactness of the related complexes. For oligomerization of BCR TM complexes, the cutoff was increased to −800 kJ/mol. Here, in order to exclude less compact complexes, an additional cutoff criterium was employed for the buried surface area (BSA) between two BCR complexes (> 10% of the total protein surface).

Protein-protein binding interfaces were assessed by analysis of the average minimum distances between all interchain residue pairs during the last 50 ns of simulation time of all CG-simulations belonging to specific binding modes and visualized in contact maps. To that end, all simulation frames from the last 50 ns of those simulations showing a compact dimer at the end of the simulation were assigned to the different dimer configuration labels (i.e., the different binding modes) using a watershed transform (56) as described in detail in Pluhackova et al. (30). Additionally, in order to pinpoint residues that contribute most to direct helix-helix interactions, the average relative interaction energy contribution (sum of Lennard-Jones and Coulomb interactions) per residue during the last 50 ns of simulation time was computed (interaction-energy profiles).

## 3. Results

Modeling of the TM domain of IgM-BCR (compare Figure 1) was addressed in spontaneous association simulations of its parts embedded in model 1-palmitoyl-2-oleoyl-sn-glycero-3-phosphocholine (POPC) bilayers. The association of the IgM-BCR TMD was investigated via extensive molecular dynamics simulations in three steps: First, the mIgM-TMD assembly was explored by analysis of the spontaneous dimerization of two copies of a μ TM heavy chain (named μ-1 and μ-2; Table 2, Step 1). Second, the spontaneous formation of the Ig-α/Ig-β-TMD heterodimer was studied (Step 2). Third, the assembly of the full IgM-BCR-TMD was explored based on the dimers obtained in the first two steps (Table 2, Steps 3a and 3b). This sequential approach relies on the following three main assumptions: (i) The individual IgM-, Ig-α-, Ig-β-domains adopt an α-helical conformation both isolated and as part of the BCR complex (7). α-helices are the predominant structural motif to span the membrane hydrophobic core (57). (ii) The BCR TMD complex is formed of one mIg-dimer and one Ig-α/Ig-β heterodimer, as previously experimentally shown [1:1 stoichiometry (7, 8)]. (iii) mIgM TM domains and Ig-α/Ig-β-TM helices pre-assemble before formation of the full IgM-BCR TM complex. The latter assumption is supported by the observed association of Ig-α/Ig-β heterodimers to mIg-dimers but not to monomers (58), and the reported disulfide bonds between Ig-α and Ig-β adjacent to the membrane (5, 59) suggesting a close proximity and preassembly of Ig-α/Ig-β TM domains.

While the assembly of transmembrane domains was analyzed from a large number of coarse-grained MD simulations, the stability of obtained quaternary structures was further studied in atomistic simulations (Table 2, Step 4). The lateral partitioning of obtained IgM-BCR TM configurations to different membrane domains was addressed at coarse-grained resolution for a membrane with both ordered and disordered domains (Table 2, Step 5). Finally, we explored the dimerization/oligomerization of IgM-BCR TMDs (Step 6) and the stability of a IgM-BCR tetramer (Step 7).

### 3.1. Assembly of mIgM-TMD Homodimer

**Figure 2:**
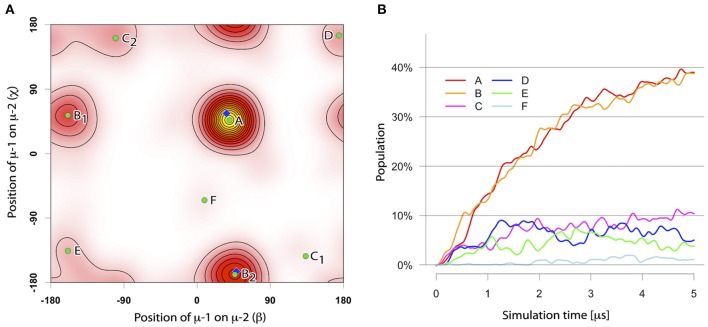
**(A)** The distinct binding modes of the mIgM-TMD assembly described by ORIANA and defined by β and χ-angles. β describes the position of monomer μ-2 with respect to monomer μ-1. χ describes the binding site of monomer μ-1 on monomer μ-2. For details please see (30, 34). Green dots mark the peaks of the bound conformers. Blue diamonds mark the β- and χ-angles of the selected representative structures for BM-A and BM-B conformers. **(B)** Population of different TM dimer configurations as a function of simulation time.

**Figure 3:**
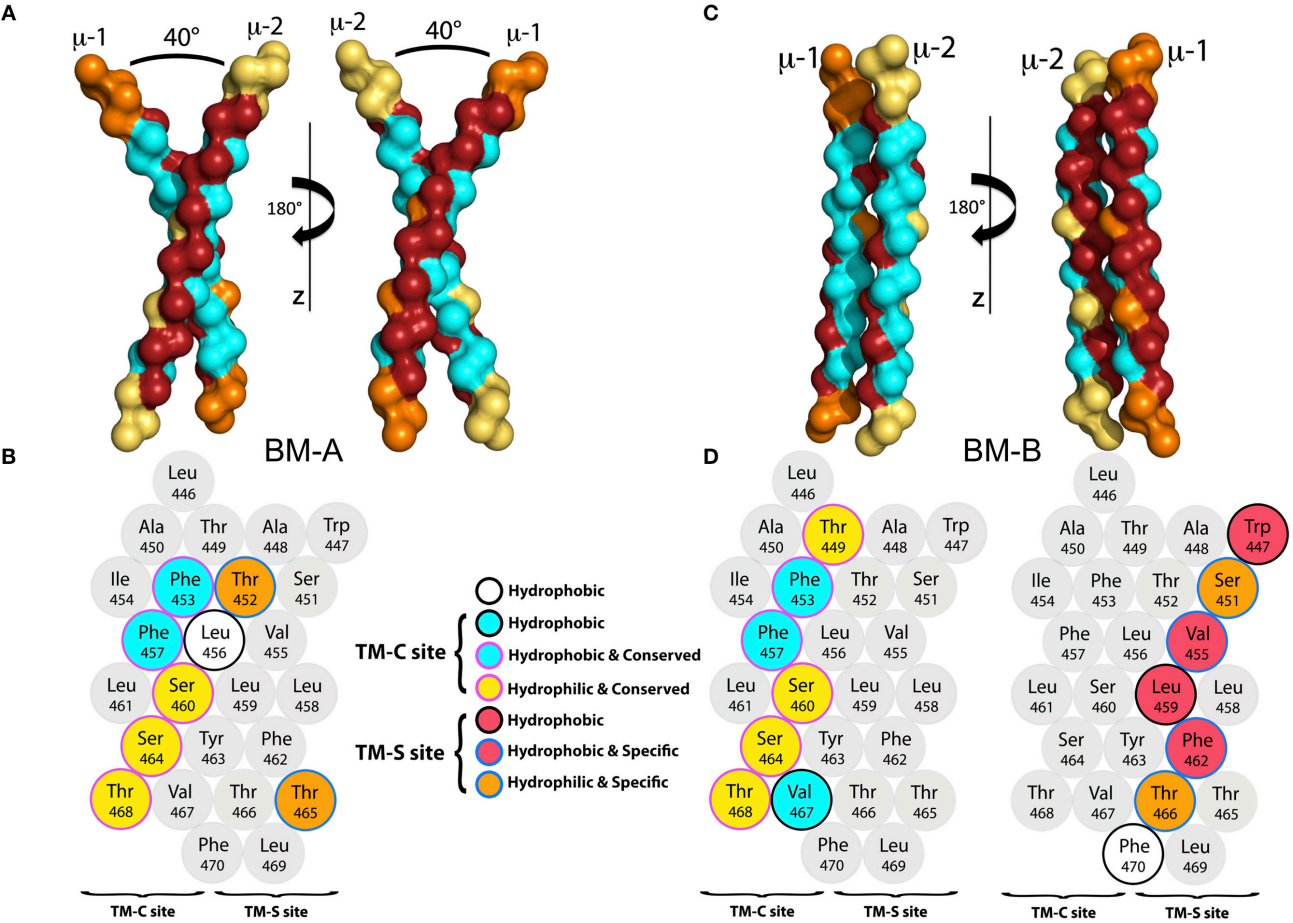
**(A)** The backbone of the representative structure of the mIgM dimer conformer BM-A in surface-representation from two different perspectives. The conserved (TM-C) sites are colored in cyan, while the specific (TM-S) sites are colored in dark red. For clarity, amino acids that neither belong to the TM-C nor to the TM-S sites are colored orange for μ-1 and yellow for μ-2. **(B)** α-helical scheme of the μ heavy chain TMD. Residues that play a key role for BM-A formation are highlighted. **(C)** Backbone structure of a representative structure of the BM-B conformer in surface-representation from two different perspectives. The TM-C sites are colored in cyan, while the TM-S sites are colored in dark red. For clarity, amino acids that belong to neither the TM-C nor to the TM-S sites are colored orange (μ-1) and yellow (μ-2). **(D)** α-helical schemes of the μ heavy chain TMDs. Residues that play a key role for BM-B formation are highlighted for μ-1 and μ-2.

The assembly of two TM μ chains modeled in α-helical conformation was studied from in total 104 simulations of each 5 μs length, starting from two monomers (μ-1 and μ-2) initially separated by 5 nm. During the spontaneous self-assembly, μ-1 and μ-2 dimerized in 102 of 104 simulations within 5 μs of simulation time (Figure 2). An orientation analysis (ORIANA) (30, 31, 34) revealed six distinct binding modes. The two dominant binding modes comprised each about 40% of the observed dimers at the end of the simulations (named BM-A and BM-B, see Figure 3). The following analysis focuses on these two major binding modes (for abbreviations of sampled binding configurations see Table 3).

**Table 3:** Dimer/oligomer configurations sampled in MD simulations and their abbreviations.

**Molecules**	**Configuration**	**Abbreviation**
mIgM homodimer	Symmetric homodimer	BM-A
	Asymmetric homodimer	BM-B
Ig-α/Ig-β	Heterodimer	BM-α
IgM-BCR TM domain	mIgM symmetric homodimer	BM-A-1, BM-A-2
	& Ig-α/Ig-β heterodimer	
IgM-BCR TM domain	mIgM asymmetr. homodimer	BM-B-1, BM-B-2
	& Ig-α/Ig-β heterodimer	

*Binding mode BM-A*. BM-A describes a symmetric, right handed homodimer of the μ TM domains. The monomers are tilted by ≈ 40^o^ relative to each other, with a tilt angle of ≈20^o^ between the membrane normal and each μ-chain. The dimerization interface comprises the central part of the TM-C sites (conserved) of each monomer, whereas the TM-S sites (specific) are turned away from the interaction site and remain freely accessible (representative structure shown in Figure 3A). A contact map analysis based on all configurations sampled for this binding mode identifies residues Thr452, Phe453, Leu456, Phe457, Ser460, Leu461, Ser464, Thr465, and Thr468 as the main contributors to helix-helix association and the major interface-forming residues of the highly symmetric interface (see Figures S1A, S2). Thereby, except for Thr452 and Thr465, the interface is dominated by conserved sites, i.e., a TM-C/TM-C dimer interface is formed. Interestingly, besides of two phenylalanines and two leucines, several hydrophilic amino acids (colored *yellow* in Figure 3B) located within the central part of the TMDs are buried at the interface. While the central regions of the TMDs are in close contact, the intracellular and extracellular termini do not associate due to the relatively large tilt of the monomers.

*Binding mode BM-B*. In the second, asymmetric binding mode, the two helices assemble in a parallel fashion (Figures 3C,D), with a dimer tilt of 25° relative to the membrane surface normal. The interface of BM-B is roughly built by the TM-C site of μ-1 and the TM-S site of μ-2. Consequently, one TM-S as well as one TM-C site are freely accessible on the surface of the mIgM TM dimer. Note that binding of the TM-C site of μ-1 to the TM-S site of μ-2 leads to the same dimer as binding of the TM-C site of μ-2 to the TM-S site of μ-1. For the sake of simplicity, the nomenclature introduced in Figure 3 is used throughout the manuscript to distinguish the positions of the BCR-forming μ chains.

The binding interface of BM-B is formed by Thr449, Phe453, Phe457, Ser460, Ser464, Val467, and Thr468 of μ-1, as identified by averaging of the interaction energies between both monomers over all configurations sampled within this binding mode. These amino acids also contribute significantly to the interaction energy of the two μ chains (Figure 3D, Figure S3). Interestingly, all of these seven residues are part of the conserved site of the helix (TM-C). In contrast, the interaction site on μ-2 is formed by the TM-S amino acids Trp447, Ser451, Val455, Leu459, Phe462, Tyr463, Thr466, and Phe470. Thus, the BM-B binding mode is characterized by a TM-S/TM-C binding interface.

Summing up, two binding configurations of the mIgM-TMD were observed which have the burial of polar amino acids at the dimer interface in common. The significantly larger helical interface of the asymmetric BM-B conformer as compared to the symmetric BM-A conformer (see Figure 3), indicates an increased stability of BM-B as also observed in all-atom (AA) simulations of both homodimer configurations (see below). While BM-A is a symmetric TM-C—TM-C dimer, BM-B perfectly aligned one TM-C and one TM-S site. Neither fits the symmetrical TM-S—TM-S dimer previously suggested (17). Blocking of both TM-C sites in the BM-A conformer would likely not allow for TM-C — Ig-α/Ig-β association as implicated by the observation that all mIgs bind the same Ig-α/Ig-β heterodimer. In contrast, the accessibility of one TM-C and one TM-S site in the BM-B conformer can account for Ig-α/Ig-β association in a 1:1 stoichiometry (60, 16). Additionally, the exposed TM-S site within the BM-B conformer is compatible with a class-specific membrane localization of the IgM-BCR (26).

### 3.2. Assembly of the Ig-α/Ig-β-TMD

**Figure 4:**
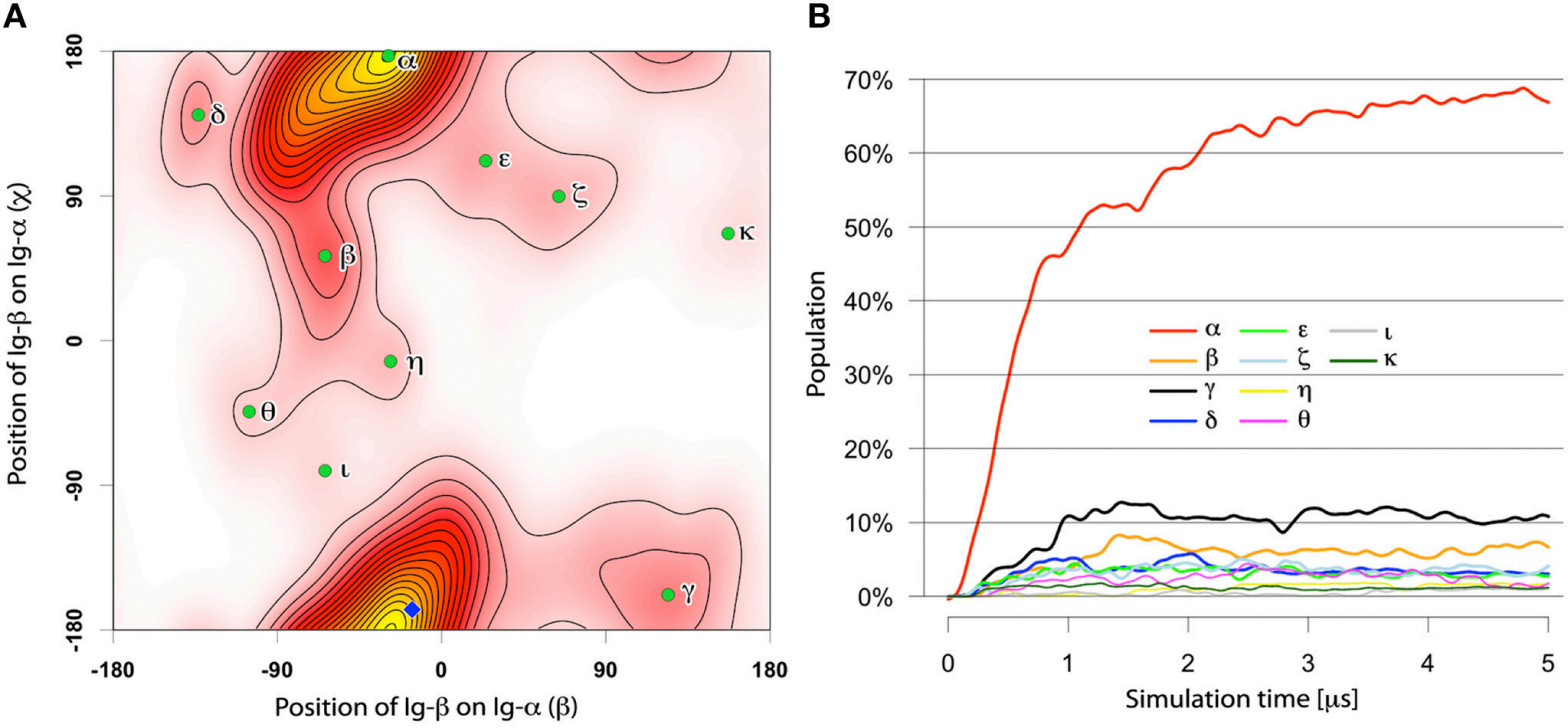
**(A)** The distinct binding modes of the Ig-α/Ig-β-TMD assembly described by ORIANA and defined by β and χ-angles. Green dots mark the peaks of the binding modes. The blue diamond marks the β- and χ-angles of the representative structures for BM-α. **(B)** Population of different Ig-α/Ig-β TM dimer configurations as a function of simulation time.

**Figure 5:**
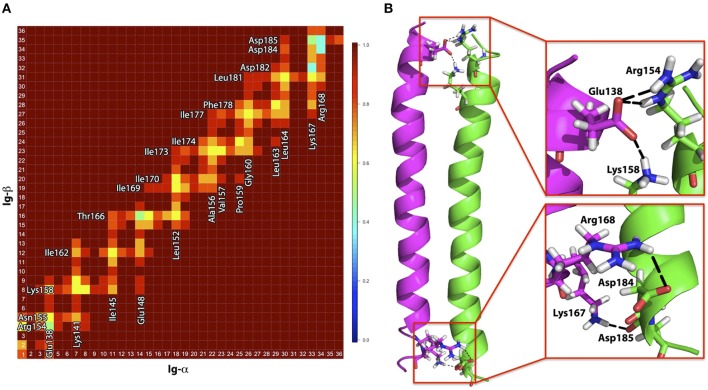
**(A)** Contact map for amino acids within the Ig-α/Ig-β dimer in the BM-α configuration. Residues in close proximity are labeled. **(B)** Representative structure of the Ig-α/Ig-β dimer (BM-α conformer) in cartoon representation. Ig-α is colored magenta, Ig-β green. Charged residues that are involved in salt-bridge formation are additionally shown as sticks and highlighted in the insets.

Of in total 105 self-assembly simulations of the Ig-α and Ig-β TM domains of each 5 μs (Table 2, Step 2), 104 systems resulted in heterodimer formation. A majority (69%) of the dimers had the same binding mode termed BM-α (Figures 4, 5). The parallel dimer is characterized by a tilt of ≈ 20° relative to the membrane normal.

*Binding mode BM-*α. The prevalent Ig-α/Ig-β dimer has a large binding interface, ranging from the intra- to the extracellular parts of the two helices and including a few charged residues located at the termini of the chains as well as a high amount of hydrophobic residues in the membrane spanning region (contact map Figure 5A). While the hydrophobic membrane-spanning residues moderately contribute to the interaction energy, high contributions were observed for the charged residues (Figure S4) forming salt bridges between the helices. In detail, Ig-α-Lys167, Ig-α-Arg168, Ig-β-Asp184, and Ig-β-Asp185 at the extracellular part of the dimer as well as Ig-α-Glu138, Ig-β-Arg154, and Ig-β-Lys158 at the intracellular part of the dimer form salt bridge networks (Figure 5B).

### 3.3. Assembly of the Full IgM-BCR-TMD

**Figure 6:**
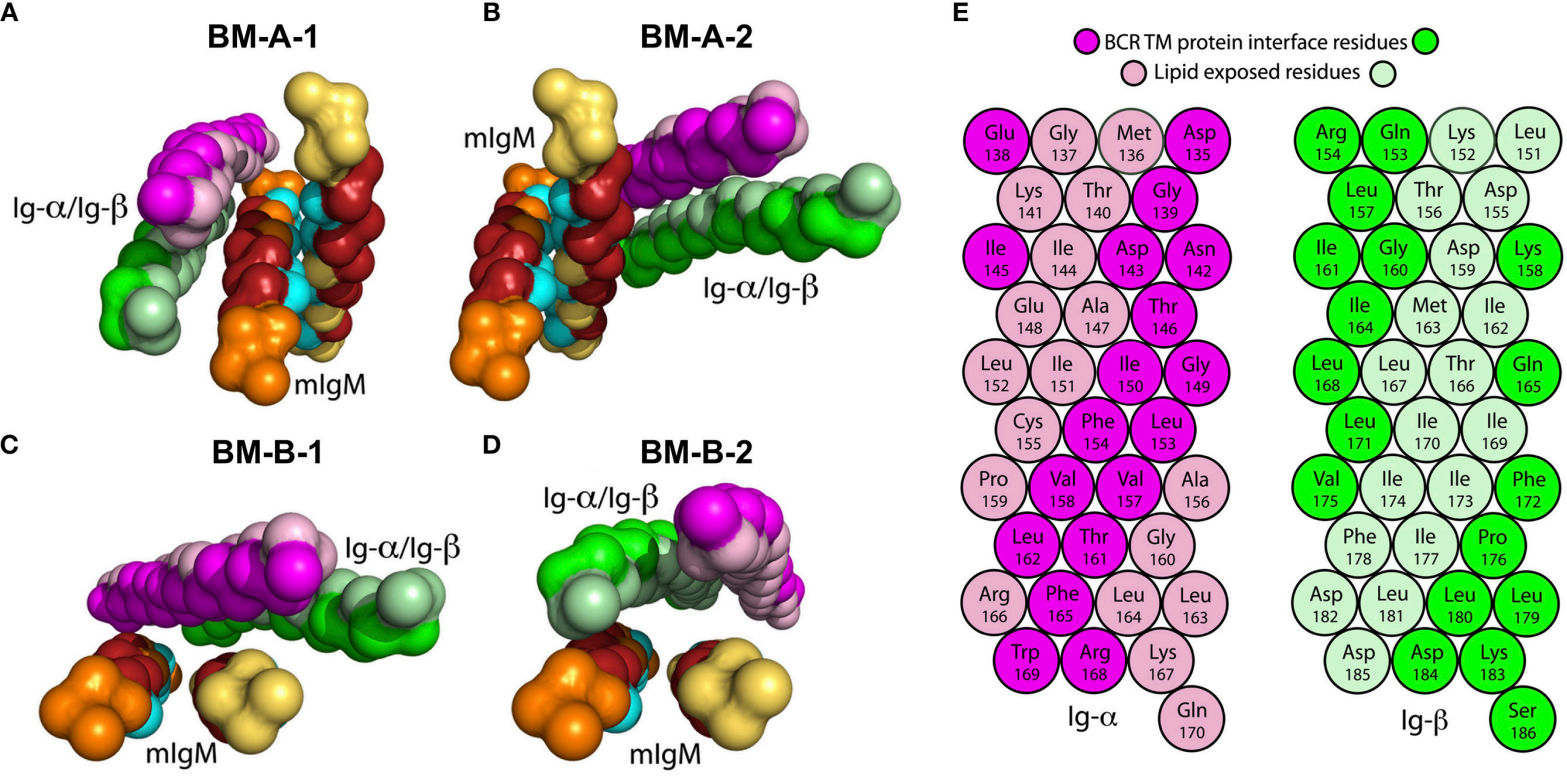
Backbone structure of assembled IgM-BCR TM domains (see Table 2, Steps 3a and 3b). The conserved sites (TM-C) of mIgM are colored in cyan, while the specific sites (TM-S) are colored in dark red. For clarity, amino acids that belong to neither the TM-C nor to the TM-S are colored orange (μ-1) and yellow (μ-2). Ig-α/Ig-β are colored in magenta and green, respectively. Residues facing the IgM interface in the BM-B-1 conformer **(C)** are shown in bright colors, lipid facing residues in light colors. **(A)** BM-A-1, **(B)** BM-A-2, **(C)** BM-B-1, and **(D)** BM-B-2 conformations. **(E)** α-helical scheme of Ig-α and Ig-β TM helices with highlighted interaction sites within the BM-B-1 tetramer.

The spontaneous association of the full IgM-BCR TM domain was studied as association of the IgM-homodimer—allowing for either of the preferred configurations (BM-A and BM-B)—and of the Ig-α/Ig-β heterodimer (BM-α) configuration (Table 2, Steps 3a, 3b). The mIgM TM dimer in BM-A conformation and the Ig-α/Ig-β heterodimer associated in 167 out of 176 simulations during 5 μs simulation time. The two main obtained tetramer configurations (binding modes BM-A-1 and BM-A-2, Figures 6A,B, respectively) were considered further. Approximately 45% of the formed tetramers belong to BM-A-1 and ≈30% of the tetramers were assigned to BM-A-2 (Figure S5). Similarly, mIgM in BM-B configuration assembled with Ig-α/Ig-β in 167 out of 190 simulations during 5 μs of simulation time. The two main tetramer binding modes were observed with a population of 25% and 21%, respectively (BM-B-1 and BM-B-2, Figures 6C,D, respectively; Figure S6).

Only the asymmetrical BM-B conformer, i.e., the tetramer configurations BM-B-1 and BM-B-2 allow for the reported 1:1 stoichiometry of mIgM and Ig-α/Ig-β (16, 60). In turn, tetramers based on the symmetric BM-A conformer (BM-A-1 and BM-A-2) could equally enable a 2:1 stoichiometry (tentative structures following a 2:1 stoichiometry are shown in Figure S7). Noteworthy, the Ig-α/Ig-β dimer is rotated by 180° in the BM-A-2 and BM-B-1 conformers as compared to BM-A-1 and BM-B-2. The orientation of Ig-α/Ig-β with respect to mIgM has implications for the association of the BCR TM domain with lipid rafts (see below).

**Figure 7:**
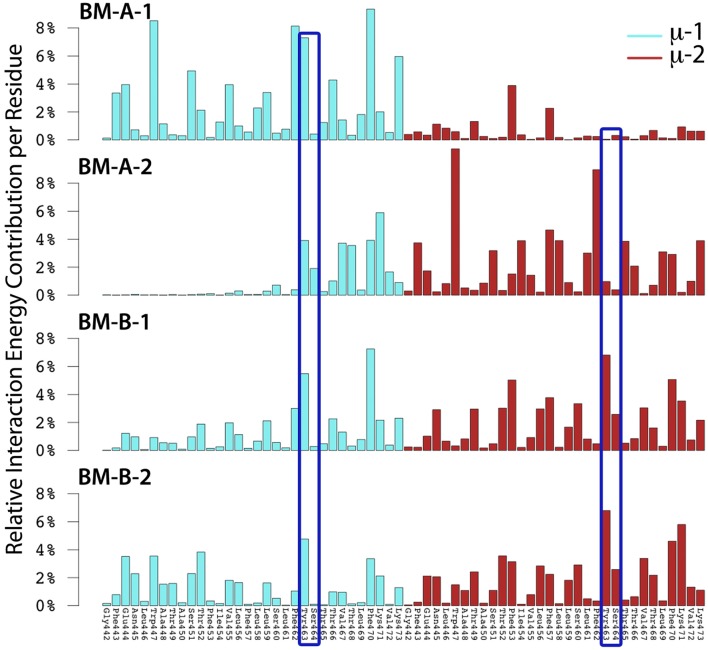
The relative interaction energy contribution of mIgM to IgM-BCR-TM domain formation for the BM-A-1, BM-A-2, BM-B-1, and BM-B-2 conformers. Residues whose mutation led to uncoupling of mIgM from Ig-α/Ig-β in experiments (10, 11, 61, 62) are highlighted by dark blue frames (Tyr463, Ser464).

Several experimental studies showed that mutation of mIgM-Tyr463 and mIgM-Ser464 to valines (YS/VV) results in uncoupling of mIgM from Ig-α/Ig-β (10, 11, 61, 62). Thus, at least one of these two residues probably plays a key role in mIgM—Ig-α/Ig-β association. While none of the two residues was shown to contribute significantly to IgM-BCR formation in BM-A-2, μ-1-Tyr463 strongly contributes to the interaction energy within BM-A-1 and μ-1-Tyr463 as well as μ-2-Tyr463 to the stability of the BM-B-1 and BM-B-2 conformers (Figure 7). Later in this study, all-atom simulations were used to show that BM-A-2, which is not stabilized by mIgM-Tyr463, does not result in a stable IgM-BCR TMD complex. These results underline the role of mIgM-Tyr463 in IgM-BCR TMD stabilization and suggest that it is the mutation of mIgM-Tyr463 and not the mutation of mIgM-Ser464 which is responsible for the uncoupling of mIgM from Ig-α/Ig-β in experiments. The IgM-BCR-TMD is additionally stabilized by the hydrophobic, aromatic residues μ-Trp447, μ-Phe462, μ-Tyr463, and μ-Phe470, which anchor within the hydrophobic TMD of Ig-α/Ig-β in all four binding modes.

### 3.4. All-Atom Validation

The employed Martini coarse-grained model may overestimate the aggregation between proteins (63), in particular for soluble proteins (64). However, highly populated dimer configurations of integral membrane proteins observed in simulation ensembles have been shown to compare well to experimental findings (30, 31, 34). Here, to identify possibly artificial configurations, atomistic simulations were used to address the stability of all self-assembled transmembrane complexes, both of dimers and of tetramers. To that end, we performed a resolution-transformation of obtained representative conformations from coarse-grained resolution to atomistic detail, employing the *backmapping* scheme (49). The conformational stability was then studied based on three 500 ns atomistic MD simulations each. All complexes were embedded in a 1-palmitoyl-2-oleoyl-sn-glycero-3-phosphoethanolamine (POPE) membrane. A POPE membrane was chosen because the thickness of the POPE bilayer at atomistic resolution resembles the thickness of the 1-palmitoyl-2-oleoyl-sn-glycero-3-phosphocholine (POPC) membrane at coarse-grained (CG) resolution used in the self-assembly simulations (compare Table S1 for data on the membrane thickness of all investigated systems). Thereby, a comparable hydrophobic thickness was achieved between atomistic resolution simulations and CG simulations.

All studied TM dimers were stable on the studied timescale with root mean square deviations (RMSD) of approximately 3 – 4 Å (Figure S8). A comparison for the mIgM dimers reveals an enhanced stability of the asymmetric BM-B configuration that is probably related to the significantly larger interface area of the bound mIgM monomers in the asymmetric configuration of 13.7 nm^2^ as compared to the symmetric structure (11.3 nm^2^).

For the full mIg-BCR TM helical tetramers, the configurations based on the asymmetric mIgM TM dimer (BM-B conformer) were stable with RMSD values between 3Å and 4Å. In contrast, the tetramers based on the symmetric mIgM TM homodimer (BM-A-1, BM-A-2) were found to be overall less stable on the 500 ns timescale with RMSD values of 4−6 Å (Figure S9). Overall, our results suggest an increased stability of IgM-BCR TM domains that contain the mIgM TM homodimer in an asymmetric configuration with a TM-S/TM-C binding interface as compared to a symmetric TM-C/TM-C interface.

### 3.5. Protein-Lipid Interactions

The association of the mIg-BCR TM complex to differently ordered membrane domains was addressed by placing the BCR transmembrane domain into a membrane consisting of a three component lipid mixture composed of 1,2-dipalmitoyl-sn-glycero-3-phosphocholine (DPPC)/1,2-di-(cis-cis-cis-9,12,15-octadecadienoyl)-sn-glycero-3-phosphocholine (DIPC)/cholesterol. This mixture is based on a three component lipid mixture of Risselada and Marrink (65) that contains a double unsaturated 1,2-di-(cis-cis-9,12-octadecadienoyl)-sn-glycero-3-phosphocholine (DUPC) as the polyunsaturated lipid and phase separates well at 295 K. In order to be able to study phase separation at 310 K, an additional C4 bead was added here mimicking an additional unsaturated bond (topology provided in the Supplementary Material). For each of the four obtained BCR TM domains, ten simulations of each 2 μs were performed at CG resolution starting from a randomized mixture of lipids within the bilayer.

**Figure 8:**
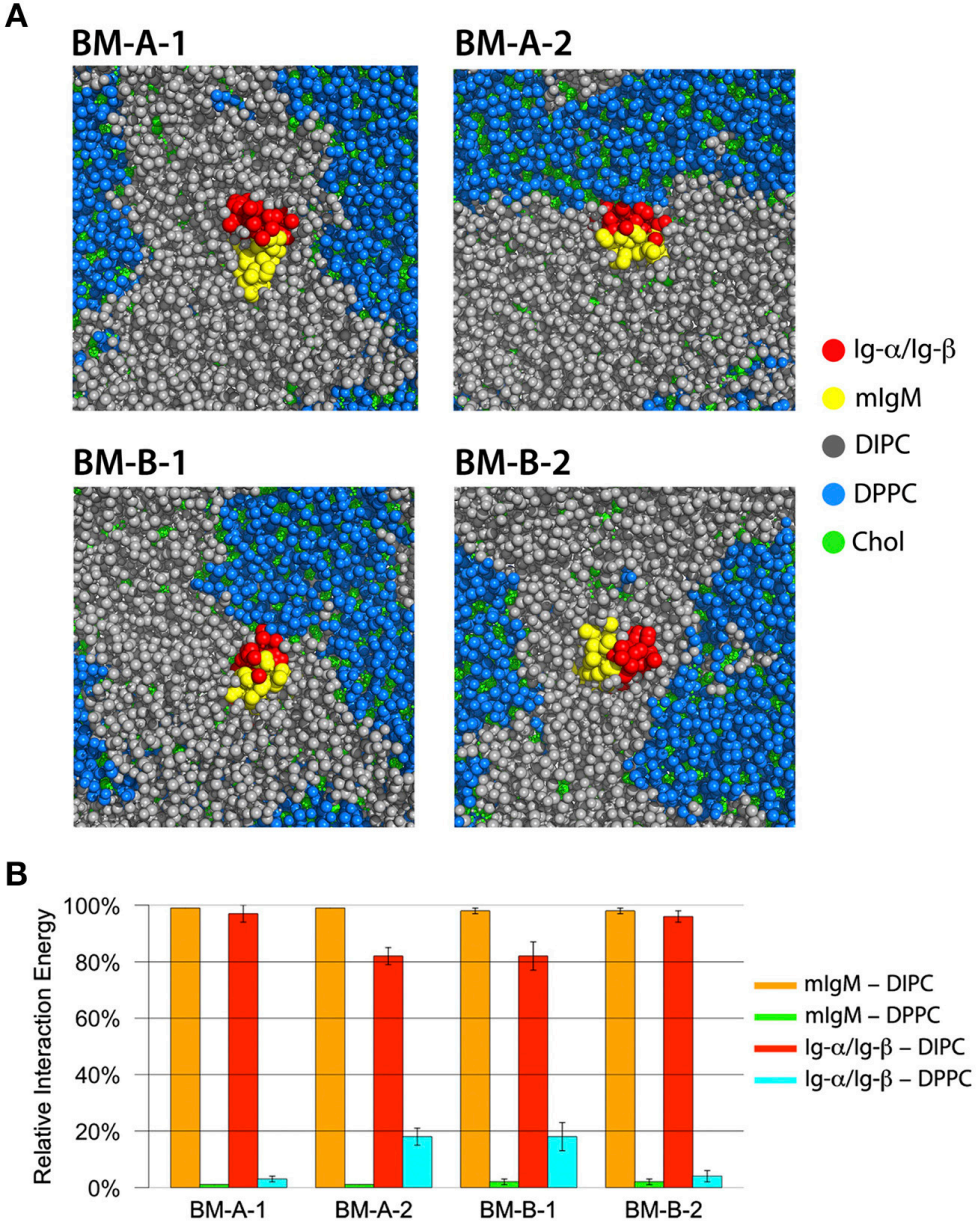
**(A)** Bottom view of representative structures of BM-A-1, BM-A-2, BM-B-1, and BM-B-2 BCR TMD models inside the DIPC/DPPC/Cholesterol membrane after 2000 ns of simulation time. The mIgM dimer is shown in red, the Ig-α/Ig-β in yellow, DIPC in gray, DPPC in blue and cholesterol in green. All molecules are shown in sphere representation. **(B)** Relative interaction energies between BCR-TMDs and DIPC/DPPC during the last 500 ns of simulation time. The error bars indicate standard error obtained from 10 individual simulations.

Within hundreds of nanoseconds of simulation time, two distinguishable lipid-phases emerged: a DPPC-rich region with a high amount of cholesterol (blue/green in Figure 8) and a DIPC-rich phase with a significantly lower amount of cholesterol (gray/green). While the DPPC/cholesterol domain assumes a liquid-ordered, raft-like phase, a fluid-disordered phase was observed for the DIPC/cholesterol domain (65). The four tetramers showed different association preferences for the liquid-disordered and -ordered membrane phases: The BM-A-1 and BM-B-2 BCR TM structures were mainly associated with the liquid-disordered phase. Differently, the BM-A-2 and BM-B-1 conformers showed a strong preference for the domain boundaries, exposing the lipid-accessible parts of their Ig-α/Ig-β-domains to the raft domains (Figure 8) as reflected by the relative interaction energies of the BCR TM conformers with the different lipid species (Figure 8B). While the lipid-accessible part of the Ig-α/Ig-β-domain is identical in BM-A-2 and BM-B-1, this part of the dimer is oriented toward mIgM in the BM-A-1 and BM-B-2 conformers (Figure 6). Thus, the lipid-exposed surface of the Ig-α/Ig-β dimers could be associated with the differential preference of the studied BCR TMD conformers to ordered or disordered membrane domains.

Only the BM-A-2 and BM-B-1 BCR conformers are compatible with the finding of Lillemeier and Mattila (25, 66) that active single BCRs are associated with lipid-raft-like domains. Of these, the BM-A-2 complex was unstable in atomistic simulations. Moreover, this conformer can't explain the importance of Tyr463 and Ser464 for coupling of mIgM with Ig-α/Ig-β and does not explain the 1:1 stoichiometry between mIgM and Ig-α/Ig-β. The latter experimental findings together with our simulation results thus provide strong support for the hypothesis that the BM-B-1 conformation represents a realistic structural model for the BCR transmembrane domain.

### 3.6. Assembly of BM-B-1 Tetramers Into Oligomers

**Figure 9:**
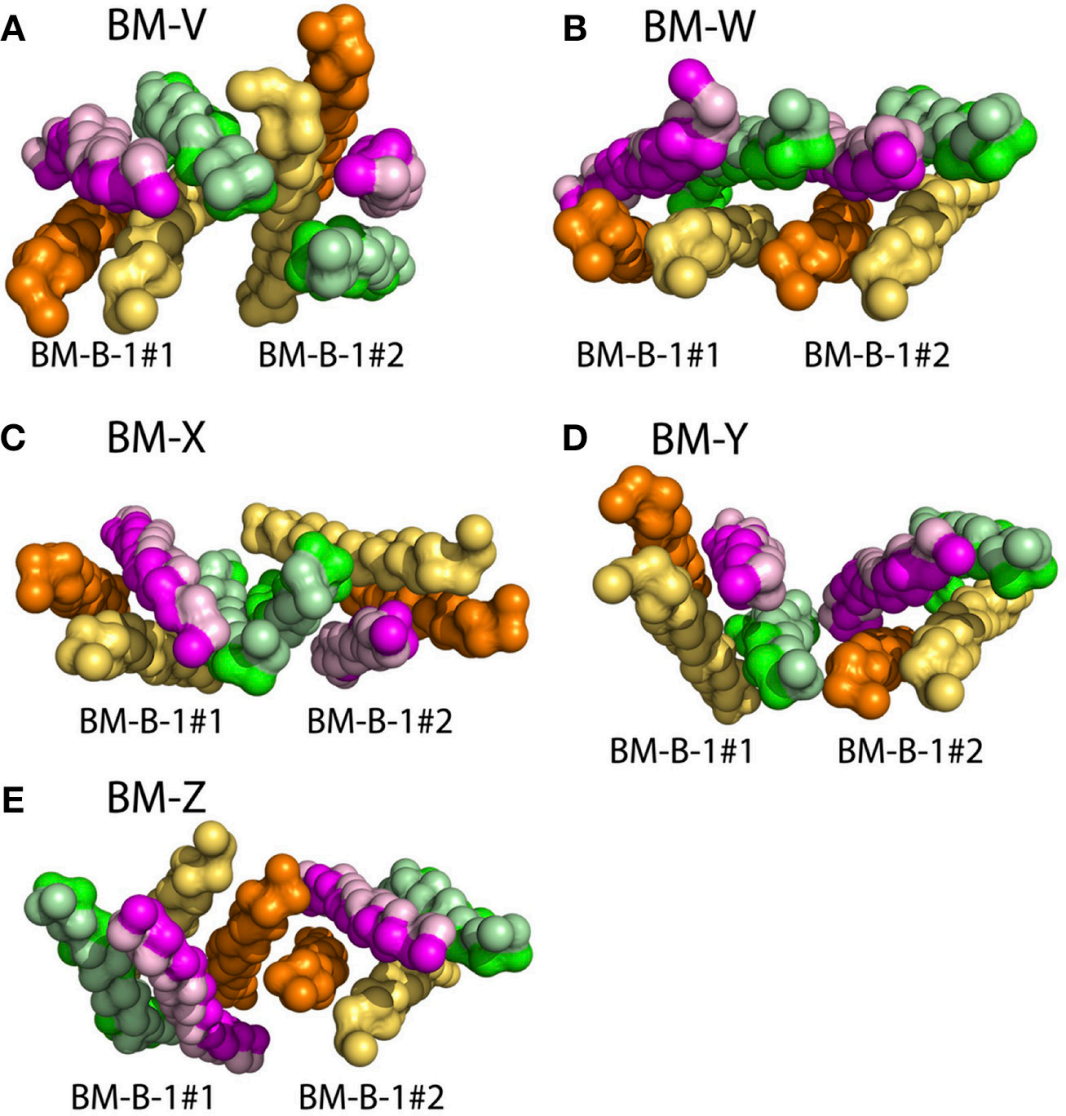
BCR TMD dimer structures as obtained from spontaneous assembly of two BCR TMDs (BM-B-1 conformation, see Table 2, Step 6). The BCRs are colored comparable to Figure 6, however the TM-C and TM-S sites of mIgM are not highlighted here. **(A)** BM-V, **(B)** BM-W, **(C)** BM-X, **(D)** BM-B-Y, **(E)** BM-B-Z conformations. See Figure S10 for orientation analysis and populations of the BCR TMD dimers.

The association of isolated BCR TM domains to ordered lipid raft domains was seen to be driven by the lipid-exposed surface of the Ig-α/Ig-β domain (see above). In turn, BCR clusters were experimentally shown to not associate with lipid rafts (25, 66). The Ig-α/Ig-β domains thus will likely be shielded from surrounding lipids upon BCR oligomerization. As a first step of oligomerization, we here addressed the spontaneous dimerization of BCR TMDs (BM-B-1 conformation) in CG simulations. The BCR TMD monomers associated in 28 of 110 simulations during 10 μs simulation time (Figure S10). Five preferred binding modes could be distinguished (BM-V, BM-W, BM-X, BM-Y, and BM-Z, see Figure 9). None of the obtained dimers fully blocked the lipid exposure of Ig-α/Ig-β.

However, the dimer structures allow for the construction of higher order oligomers. For example, a symmetric BCR tetramer built based on the BM-Y binding mode (see Figure 9) shields all four Ig-α/Ig-β domains from the surrounding lipid environment (Figure S11). This cluster was found to be stable on the 500 ns timescale at atomistic resolution (Figure S12). Ig-α/Ig-β may as well be shielded in a hexameric configuration (Figure S13). This differential lipid accessibility suggested by the lipid-exposure of Ig-α/Ig-β in BCR monomers and their possible burial in higher order BCR oligomers provides a natural explanation for the observed shift in the BCR lipid environment after activation-induced BCR cluster opening (26).

## 4. Discussion

**Figure 10:**
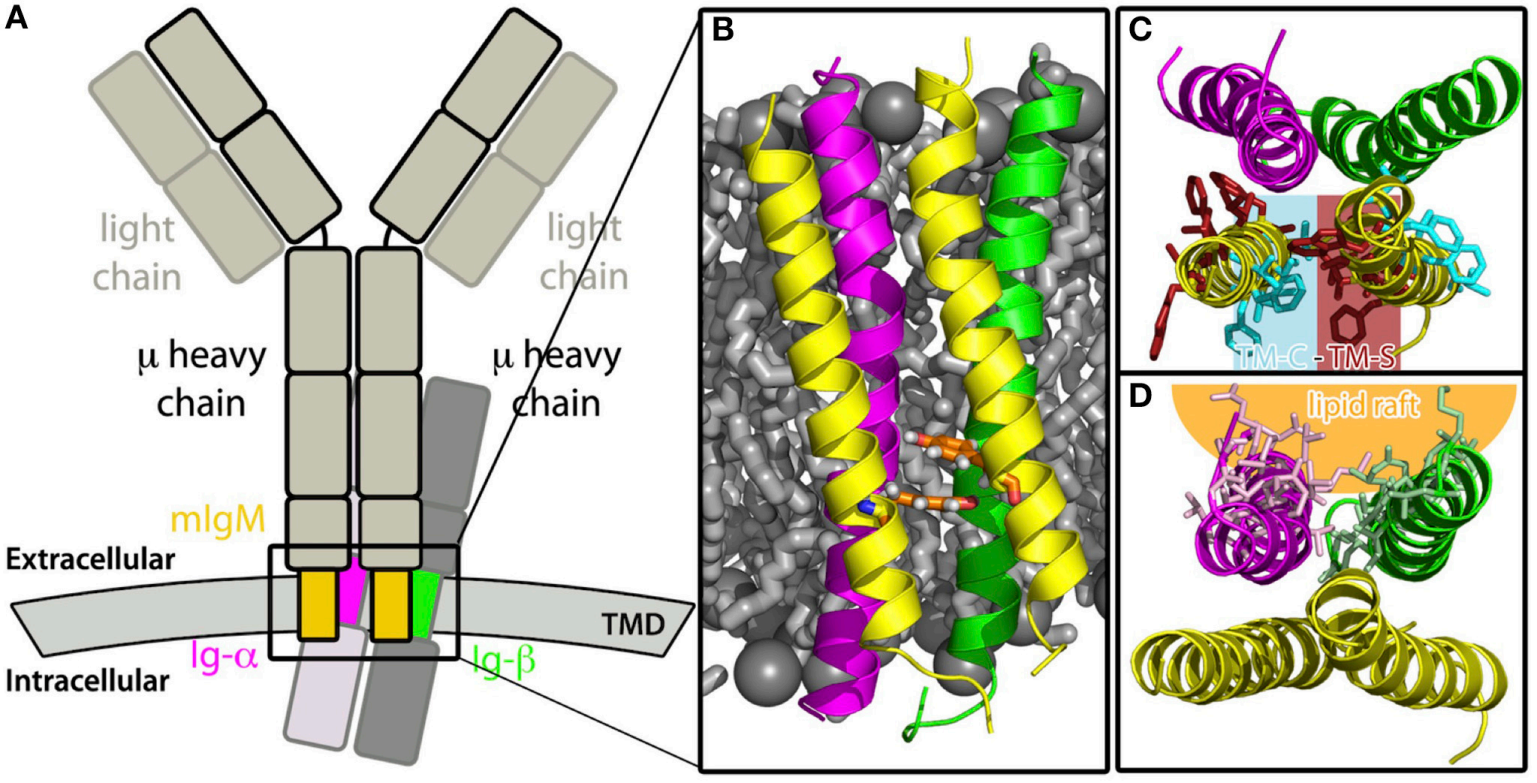
Summary. **(A)** Sketch of a monomeric IgM-BCR. The transmembrane domains, whose assembly was studied here, are highlighted in color, namely the TMDs of the μ chains are colored yellow, Ig-α magenta, and Ig-β green. **(B)** Front view of the modeled IgM-BCR transmembrane domain embedded in a POPE membrane (shown as gray sticks and spheres). The protein helices are shown in cartoon representation and the coloring corresponds to subfigure A. Tyr463 of both μ heavy chains which interact with Ig-α/Ig-β, thus strongly stabilizing the IgM-BCR TMD, are highlighted by orange sticks. Other side chains were omitted for clarity. **(C)** View on the IgM-BCR TMD in cartoon representation from the extracellular side. The helical transmembrane tetramer is stabilized by interactions of one TM-C with one TM-S site of the individual μ chains. Residues which were shown to play a key role in mIgM stabilization (see Figures 3C,D) are shown as sticks and colored in cyan (conserved site, TM-C), or in darkred (specific residues, TM-S site). Other side chains and hydrogen atoms are omitted for clarity. **(D)** Extracellular view on the IgM-BCR TMD in cartoon representation with highlighted residues (stick representation) of Ig-α/Ig-β, which preferably interact with lipid-raft like domains.

While many antibody and antibody/antigen structures could be resolved in the past, the arrangement of antibodies within and the overall three-dimensional structure of both the cytoplasmic BCR domain and of the BCR transmembrane domain are unknown. Here, using a combination of coarse-grained and atomistic molecular dynamics simulations, we studied the spontaneous self-assembly of the helices building the transmembrane domain of IgM B-cell receptors. The obtained conformation of the BCR TMD is characterized by an asymmetric mIgM dimer (TM-C/TM-S) bound to Ig-α/Ig-β (see Figure 10). The latter contacts both mIgM molecules, a finding that is supported by previous experiments reporting that Ig-α/Ig-β only co-purified with the mIg dimer, but not with a single heavy chain/light chain pair (58). In the favored BCR TMD structure, Tyr463 of mIgM contributes significantly to the stability of the helical tetramer (see also Figure 10B). This finding is corroborated by previous mutation analysis of these sites, a double mutation (YS/VV) led to uncoupling of mIgM from Ig-α/Ig-β (10, 11, 61, 62).

The asymmetric TM-C/TM-S mIgM TM dimer (Figure 10C) is in contrast to the symmetrical TM-S/TM-S dimer proposed earlier by Reth (17). However, as outlined in the introduction, a symmetric TM-S/TM-S dimer would probably violate the 1:1 stoichiometry of IgM:Ig-α/Ig-β (7, 8). Also, complexes built using a symmetric mIgM dimer exhibited a reduced stability in atomistic MD simulations. Ig-α – Ig-β assembly resulted in a single stable dimer configuration, which experimentally has been poorly characterized so far. Its stability stems on the one hand from salt bridges between charged residues at the intra- and extracellular parts of the Ig-α and Ig-β TMDs and on the other hand from the large hydrophobic interface built by the membrane spanning regions of Ig-α and Ig-β. We could further show that the lipid accessible part of Ig-α/Ig-β within the BCR TMD likely drives the association of these complexes with ordered lipid domains (Figure 10D) (25, 66). However, it has to be noted that monomeric BCR TMDs did not fully partition to the lipid raft like domains but rather associated to the interface between ordered and disordered domains. This is possibly coupled to a recently reported enhanced enrichment of transmembrane peptides at domain interfaces in coarse-grained simulations employing the Martini forcefield (67). However, different from the latter study, we here employed different membrane phases of similar thickness. Still, a comparative partitioning analysis for various BCR TM models differing in the orientation of the Ig-α/Ig-β dimer within the BCR revealed an interface-dependent partitioning of BCR either to the disordered membrane domain (BM-A-1, BM-B-2 conformers) or to the ordered-disordered domain interface (BM-A-2, BM-B-1). This clearly shows a protein interface-dependent membrane partitioning within the chosen coarse-grained methodology. A more detailed analysis of the driving forces for differing membrane domain associations would require to scrutinize the underlying lipid-protein interactions in ordered, disordered, and interfacial membrane domains at atomistic resolution.

Oligomer models for the BCR TMD provide cues for the mechanisms underlying the observed translocation of BCRs upon activation from non-raft to lipid raft domains (68, 69): passive IgM-BCRs may reside as oligomers with shielded Ig-α/Ig-β interfaces within non-raft regions while the oligomers may be opened or re-organized upon activation (26, 70) resulting in release of the Ig-α/Ig-β membrane interfaces and thus changed preference for lipid raft domains.

In summary, we suggest a structural model for the transmembrane domain of IgM-BCR that is in line with the available experimental data. Monomer and oligomer structures and their differing membrane domain association provide a molecular view on the dissociation activation model, which states that activation-induced BCR cluster opening leads to a transition of single, active BCRs from fluid membranes to lipid-raft like domains (26). Similar couplings between the assembly or clustering of membrane proteins on the nanoscale and signaling were reported for a number of receptors (71), e.g., for the formation of microclusters of T-cells receptors and the linker for activation of T cells (Lat) during T cell activation (72). Multiscale simulations, combining coarse-grained and atomistic MD simulations in a sequential manner (73) provide an exciting and promising tool in the study of the structure, the clustering and the domain preference of receptors at atomistic resolution.

## Author Contributions

MF and KP performed MD simulations and analysis. RB designed and supervised the study. All wrote the manuscript.

### Conflict of Interest Statement

The authors declare that the research was conducted in the absence of any commercial or financial relationships that could be construed as a potential conflict of interest.
