# Supplementary material for: Structural Model of the mIgM B-Cell Receptor Transmembrane Domain From Self-Association Molecular Dynamics Simulations
Source: Front Immunol. 2018 Dec 17;9:2947. doi: 10.3389/fimmu.2018.02947 (PMC6304377; doi:10.3389/fimmu.2018.02947)
Supplement: Supplementary file 1 [file Data_Sheet_1.pdf]

Supplementary Material:
Structural model of the mIgM B-cell Receptor Transmembrane Domain from Self-Association Molecular Dynamics Simulations

Mario D. Friess [1], **Kristyna Pluhackova** [1,2] **and Rainer A. Böckmann** [1,*]

[1] *Computational Biology, Department of Biology, Friedrich-Alexander University of Erlangen-Nürnberg, Erlangen, Germany*
[2] *Current address: Biophysics Group, Department of Biosystems Science and Engineering, ETH Zürich, Basel, Switzerland*

Correspondence*:
Rainer A. Böckmann
rainer.boeckmann@fau.de

1 SUPPLEMENTARY: MARTINI TOPOLOGY FOR DIPC

```
; DI alpha-LINOLENYL PHOSPHATIDYLCHOLINE (18:3-18:3 PC)
; or di-(cis-cis-cis-9,12,15-octadecadienoyl)-PC
;
[ moleculetype ]
; molname nrexcl
DIPC 1
[ atoms ]
;id type resnr residu atom cgnr char
1 Q0 1 DIPC NC3 1 1.0
2 Qa 1 DIPC PO4 2 -1.0
3 Na 1 DIPC GL1 3 0
4 Na 1 DIPC GL2 4 0
5 C2 1 DIPC C1A 5 0
6 C4 1 DIPC D2A 6 0
7 C4 1 DIPC D3A 7 0
8 C4 1 DIPC D4A 8 0
9 C2 1 DIPC C5A 9 0
10 C2 1 DIPC C1B 10 0
11 C4 1 DIPC D2B 11 0
12 C4 1 DIPC D3B 12 0
13 C4 1 DIPC D4B 13 0
14 C2 1 DIPC C5B 14 0
[bonds]
; i j funct length force.c.
1 2 1 0.47 1250
2 3 1 0.47 1250
3 4 1 0.37 1250
3 5 1 0.47 1250
5 6 1 0.47 1250
6 7 1 0.47 1250
7 8 1 0.47 1250
8 9 1 0.47 1250
4 10 1 0.47 1250
10 11 1 0.47 1250
11 12 1 0.47 1250
12 13 1 0.47 1250
13 14 1 0.47 1250

[angles]
; i j k funct angle force
2 3 4 2 120.000 25.0
2 3 5 2 180.000 25.0
3 5 6 2 180.000 10.0
5 6 7 2 120.000 10.0
6 7 8 2 100.000 10.0
7 8 9 2 120.000 45.0
4 10 11 2 180.000 10.0
10 11 12 2 120.000 10.0
11 12 13 2 100.000 10.0
12 13 14 2 120.000 45.0
```

2 SUPPLEMENTARY TABLES AND FIGURES

Table S1. Membrane thickness in the vicinity of the protein and in protein-distal areas in dependence of the lipid type and resolution of the MD simulations (all-atom vs. coarse-grained).
[a] The thickness of the bilayer within a radius of 1 nm around the protein
[b] The thickness of the bilayer at least 2 nm, and not more than 3 nm away from the protein.
The membrane thickness was defined as distance between the centers of mass of the phosphate atoms belonging to the lipid headgroups in the upper layer and in the lower layer. In the case of atomistic simulations, the average value for the time window between 100 ns – 200ns of the simulation was computed. For the CG complexes, the average was analyzed over the last 500 ns of all CG simulations.

**Complex**	d_p^a [nm] (AA)	d_m^b [nm] (AA)	d_p^a [nm] (CG)	d_m^b [nm] (CG)
		**POPC**		
**BM-A**	3.66 ± 0.12	3.81 ± 0.07	4.02 ± 0.01	4.25 ± 0.00
**BM-B**	3.55 ± 0.13	3.84 ± 0.09	4.01 ± 0.03	4.24 ± 0.00
**BM-α**	3.75 ± 0.11	3.81 ± 0.08	4.20 ± 0.04	4.21 ± 0.02
**BM-A-1**	3.88 ± 0.08	3.77 ± 0.10	4.12 ± 0.04	4.22 ± 0.01
**BM-A-2**	3.68 ± 0.11	3.79 ± 0.10	4.11 ± 0.04	4.22 ± 0.01
**BM-B-1**	3.83 ± 0.08	3.74 ± 0.11	4.13 ± 0.04	4.21 ± 0.01
**BM-B-2**	3.80 ± 0.10	3.73 ± 0.09	4.12 ± 0.03	4.21 ± 0.02
		**POPE**		
**BM-A**	4.03 ± 0.10	4.21 ± 0.07	-	-
**BM-B**	4.18 ± 0.10	4.16 ± 0.07	-	-
**BM-α**	4.04 ± 0.10	4.21 ± 0.07	-	-
**BM-A-1**	4.17 ± 0.07	4.23 ± 0.08	-	-
**BM-A-2**	4.03 ± 0.06	4.20 ± 0.08	-	-
**BM-B-1**	4.01 ± 0.07	4.14 ± 0.06	-	-
**BM-B-2**	4.24 ± 0.06	4.24 ± 0.06	-	-

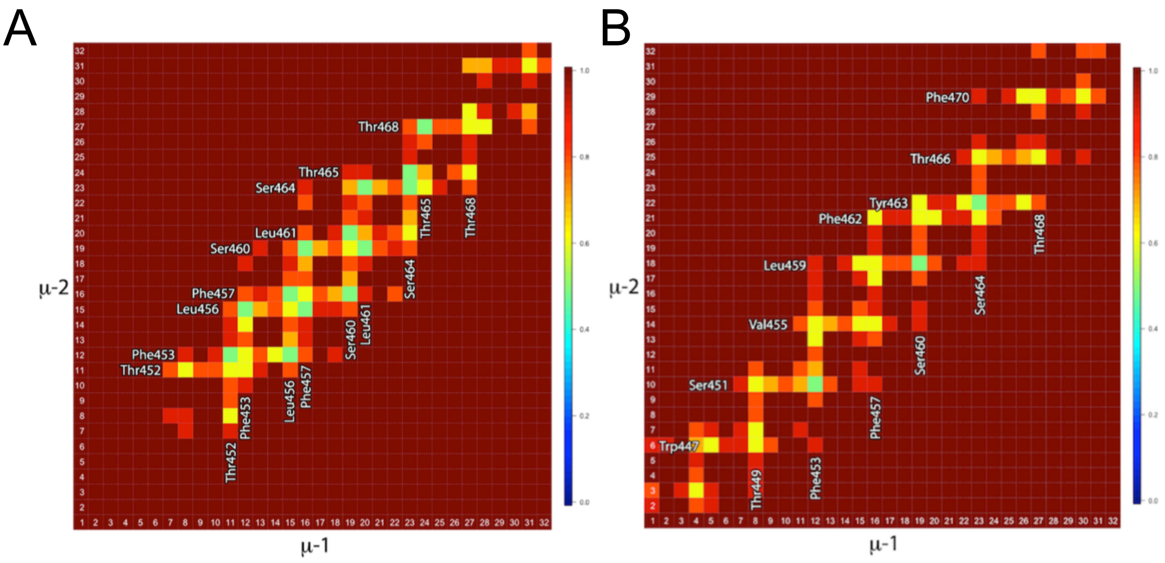

Figure S1. **(A)** Contact map of mIgM BM-A conformer. Residues in close proximity to the bound monomer are labeled.**(B)** Contact map for the BM-B conformer. Residues in close proximity are labeled.

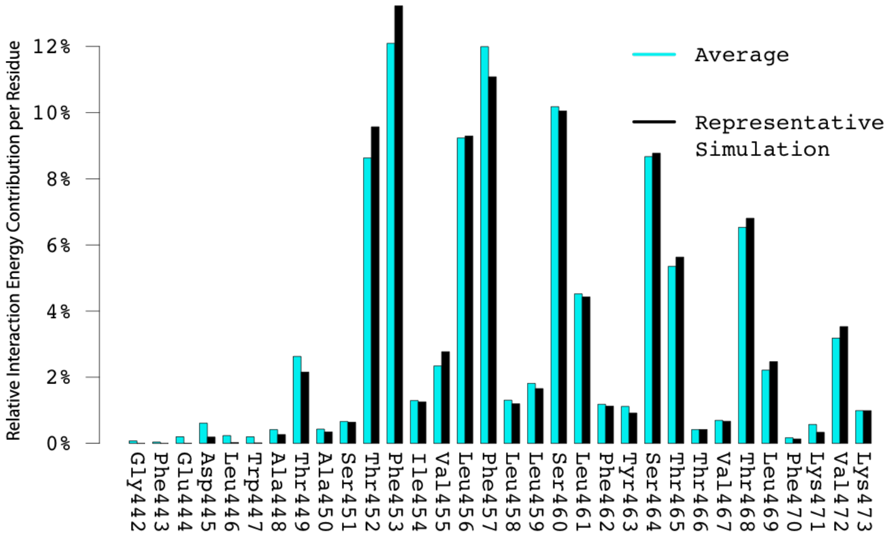

Figure S2. The interaction energy profile for the symmetric BM-A mIgM dimer conformer.

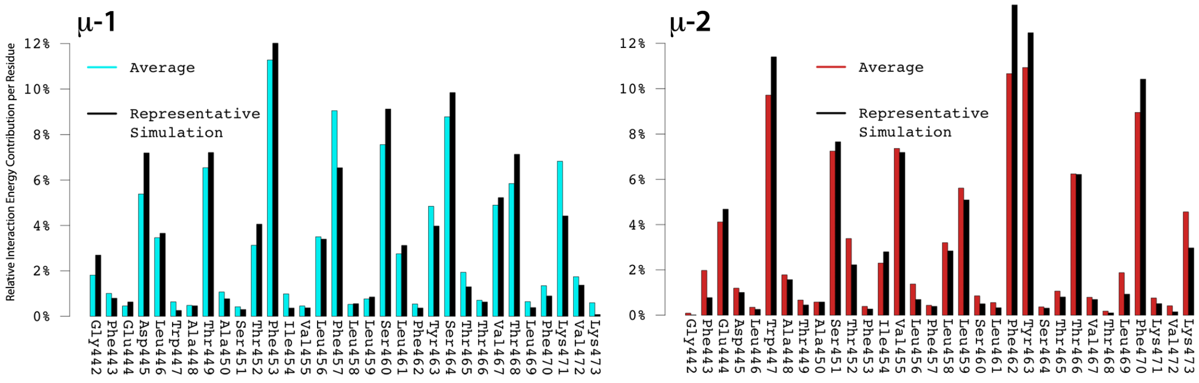

Figure S3. The interaction energy profile for the asymmetric mIgM dimer conformer (BM-B binding mode).

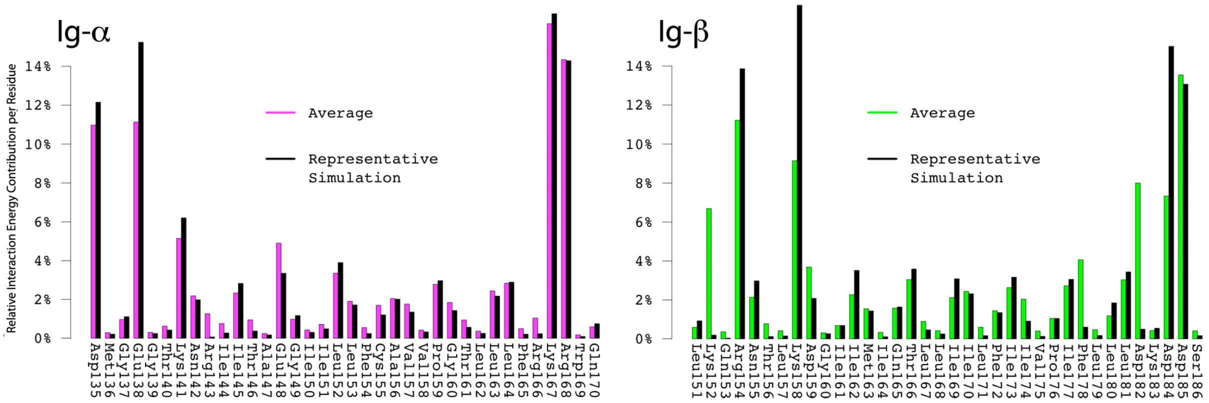

Figure S4. The interaction energy profile for spontaneously associated Ig-α/Ig-β dimers in the BM-α binding mode.

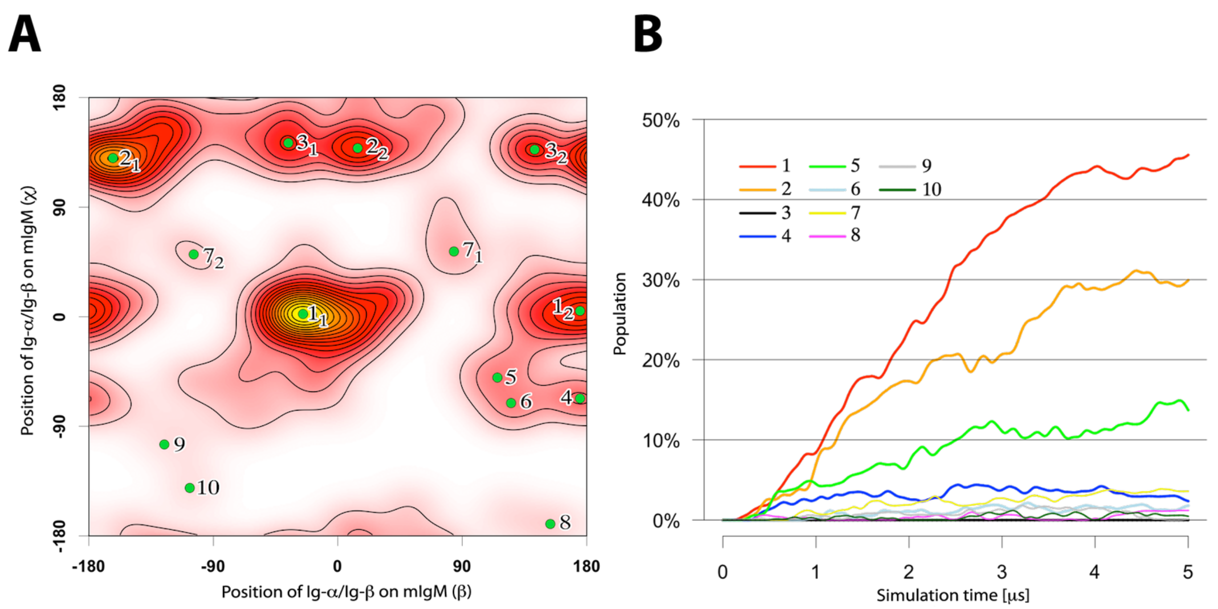

Figure S5. **(A)** The distinct binding modes of the IgM-BCR-TMD assembly involving the BM-A mIgM dimer and the Ig-α/Ig-β dimer (BM-α conformer) described by ORIANA and defined by β and χ-angles. Green dots mark the peaks of the individual binding modes. Since this DAFT setup includes one symmetrical and one asymmetrical dimer, a shift of the χ angle by 180° combined with a constant β angle in the ORIANA map describes equivalent binding modes. **(B)** Population of different IgM-BCR TM configurations as a function of simulation time.

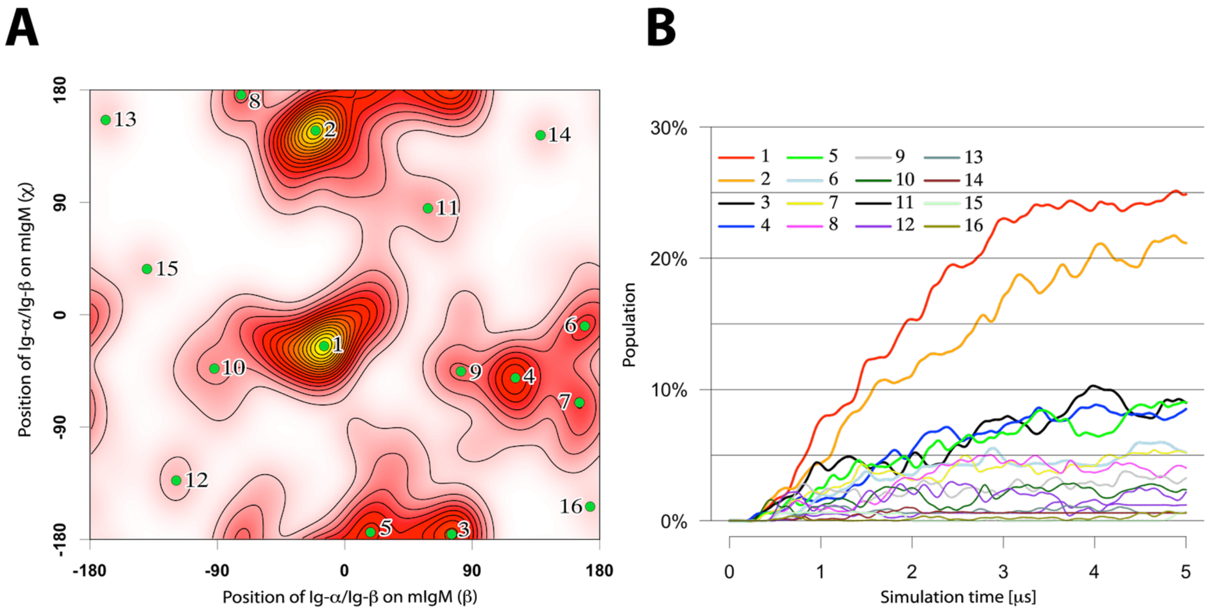

Figure S6. **(A)** The distinct binding modes of the IgM-BCR-TMD assembly involving the BM-B mIgM dimer and the Ig-α/Ig-β dimer (BM-α conformer) described by ORIANA and defined by β and χ-angles. Green dots mark the peaks of the individual binding modes. **(B)** Population of different IgM-BCR TM configurations as a function of simulation time.

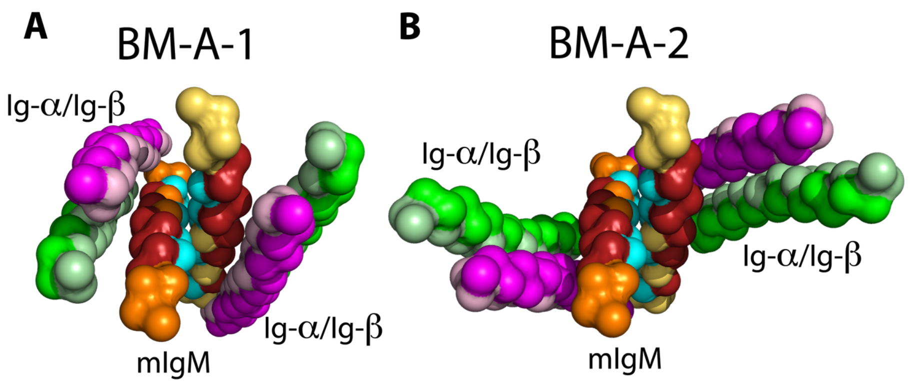

Figure S7. Visualization of the possible 1:2 stoichiometry between mIgM and Ig-α/Ig-β for **(A)** BM-A-1 and **(B)** BM-A-2 conformations of the mIgM TM dimer.

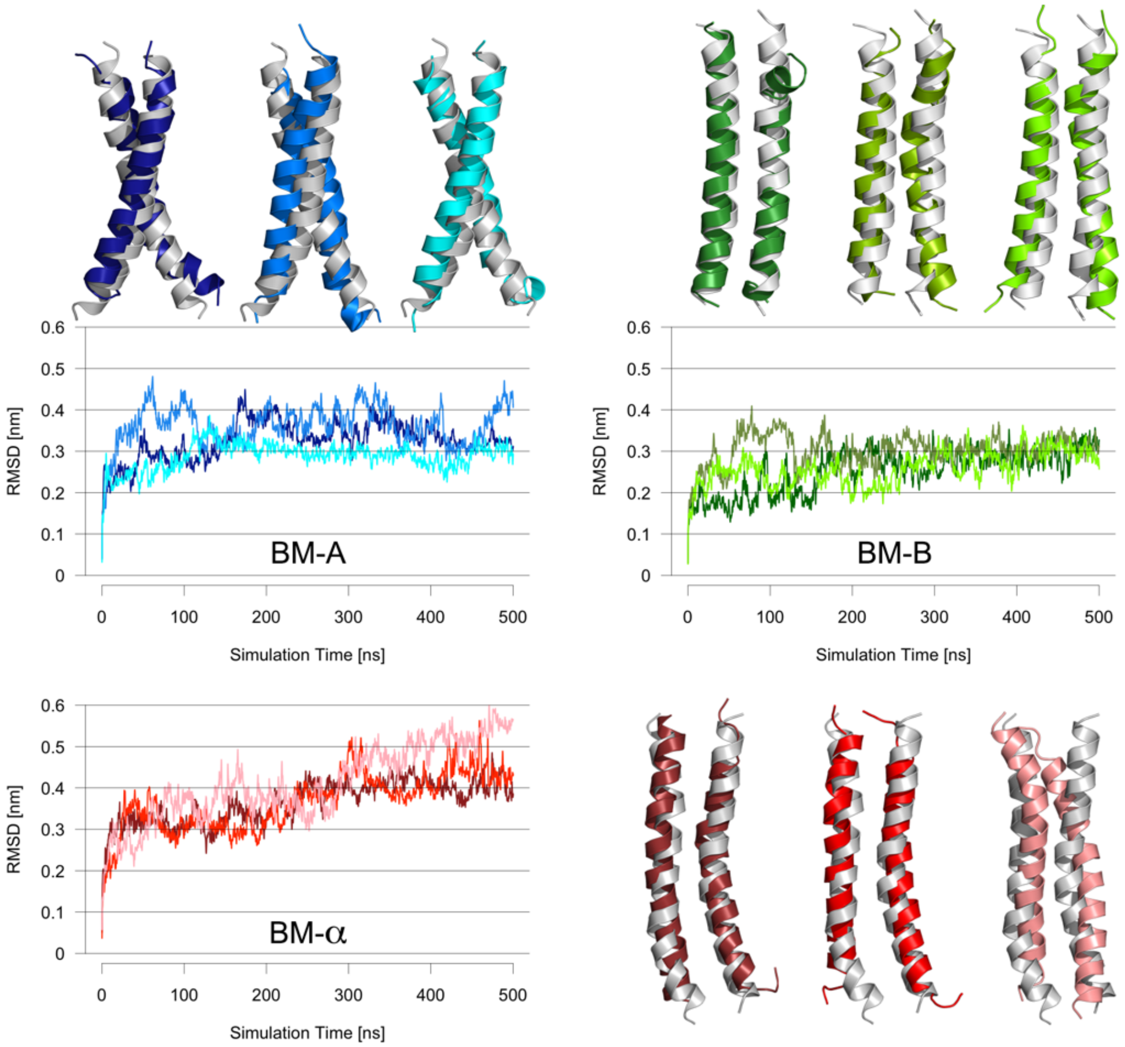

Figure S8. Overlay of starting structures (grey) of the atomistic mIgM (BM-A, BM-B binding modes) and Ig-α/Ig-β (BM-α binding mode) dimers in cartoon representation with corresponding structures after 500 ns of simulation time (coloured, three replicas each). Additionally, the backbone root mean square deviation (RMSD) of the three dimers (BM-A, BM-B, and BM-α) embedded in a POPE membrane during 500 ns of simulation time for three replicas each is shown.

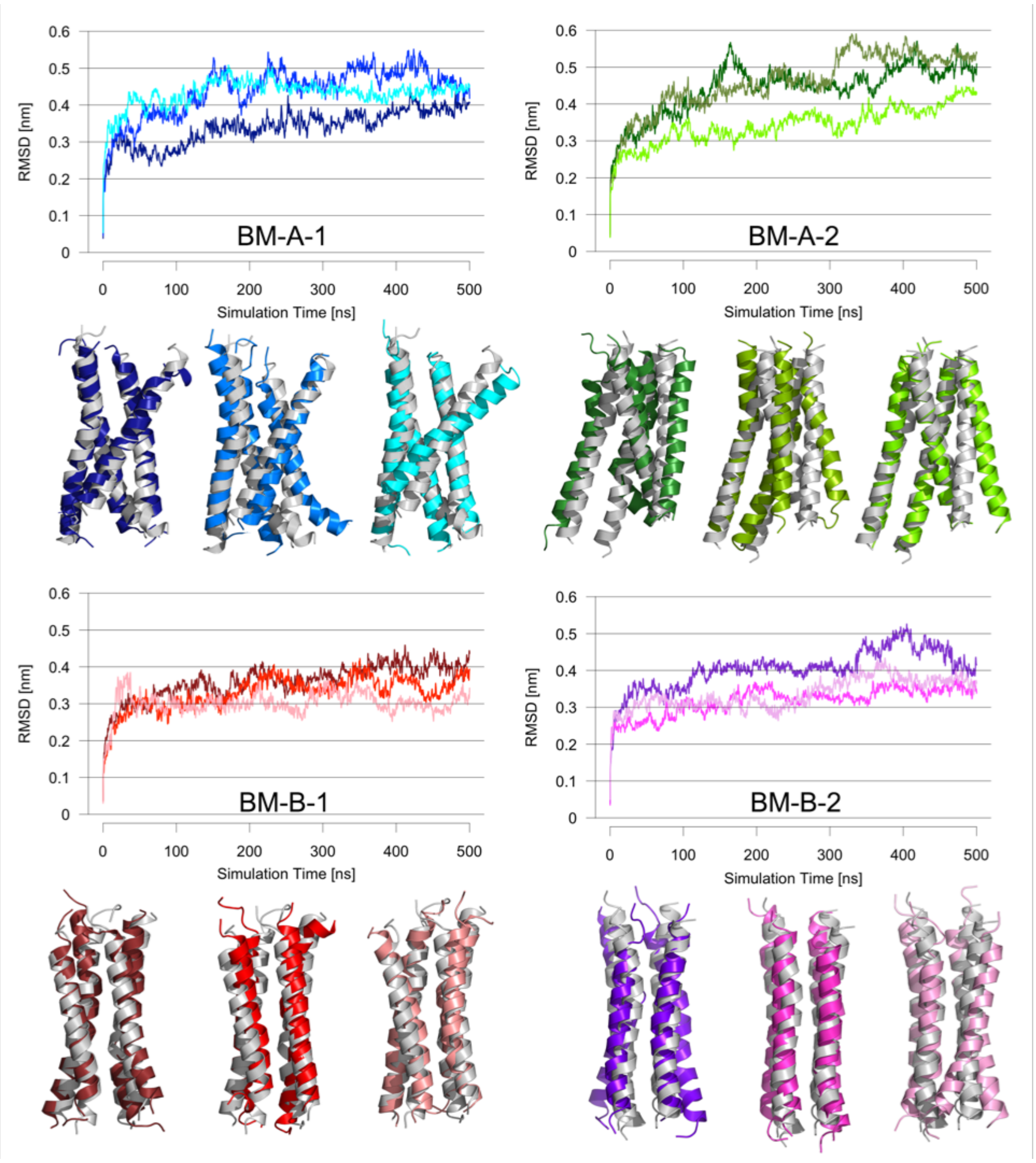

Figure S9. Overlay of starting structures (grey) of atomistic IgM-BCR TM domains in cartoon representation with the corresponding structures after 500 ns of simulation time (coloured, three replicas each). Additionally shown are the backbone RMSD values of the four tetramers (BM-A-1, BM-A-2, BM-B-1 and BM-B-2 binding modes) embedded in a POPE membrane during 500 ns of simulation time for three replicas each.

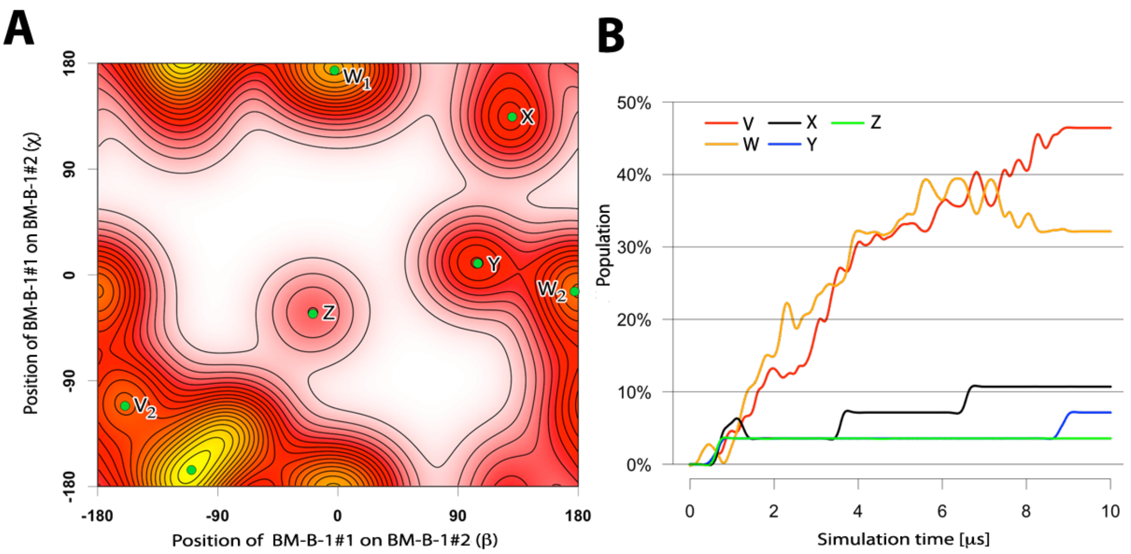

Figure S10. The distinct binding modes of the spontaneous assembly of two BCR TMDs (in BM-B-1 conformation) described by ORIANA and defined by β and χ-angles. Green dots mark the peaks of the BMs. (B) The binding mode time development of the populations during the assembly of two BM-B-1 BCRs.

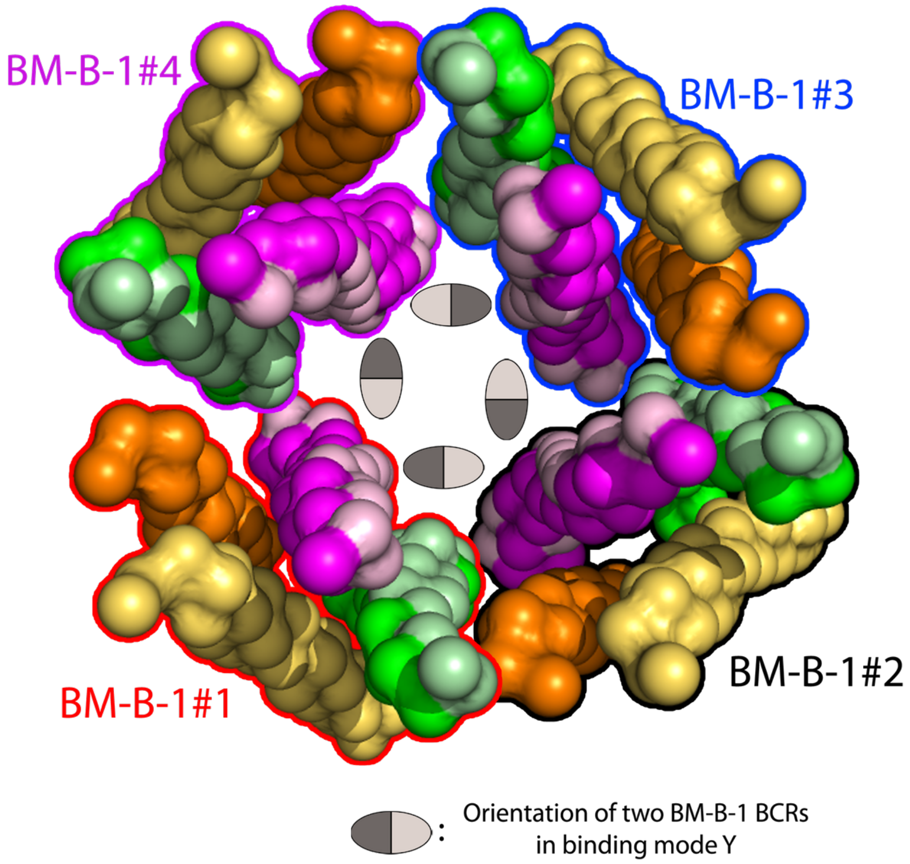

Figure S11. A tetramer composed of four BCR TM domains (BM-B-1 conformation). Starting from a BCR dimer (BM-Y conformer), the complex was extended stepwise by adding BCR TMDs. The complex was shown to be stable in atomistic simulations.

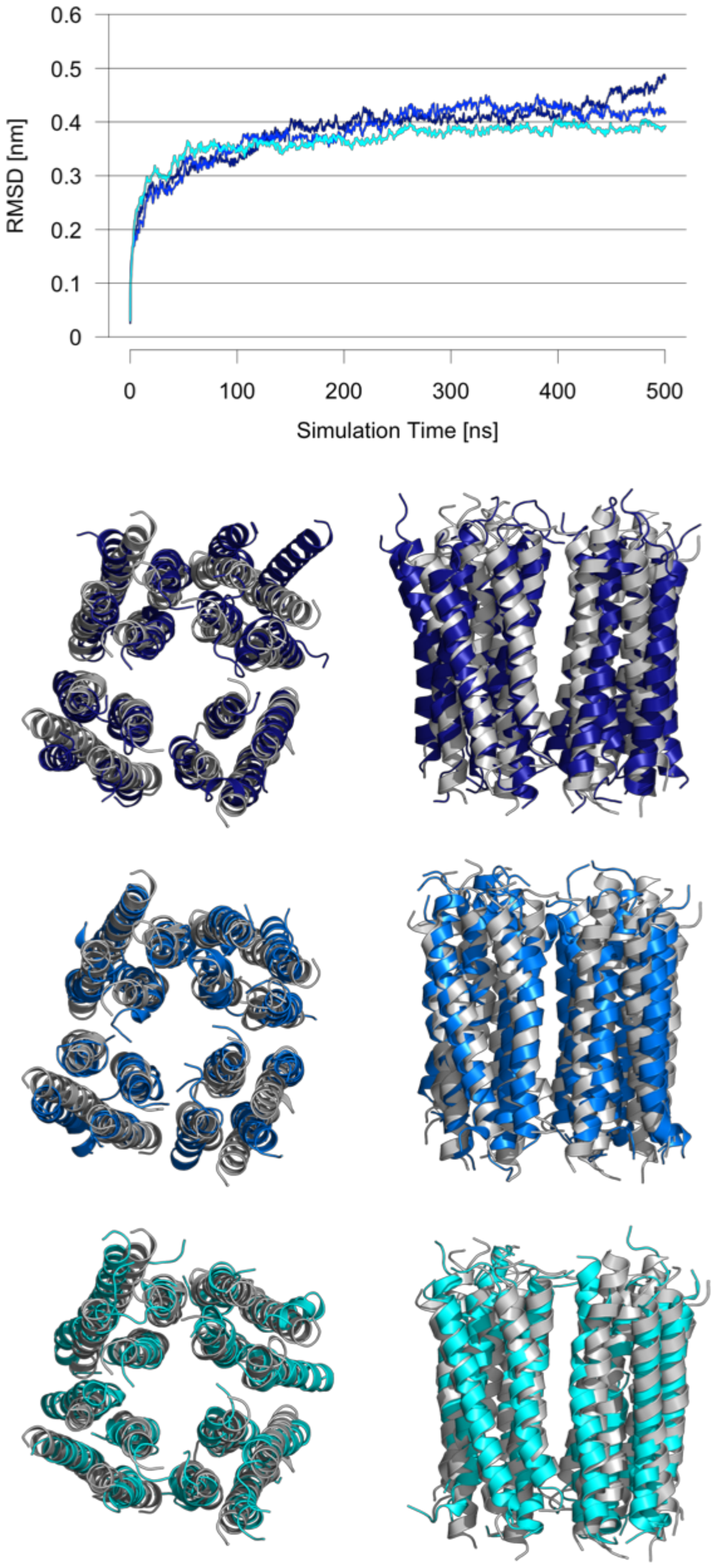

Figure S12. Backbone RMSD of the backmapped BCR TMD tetramer and comparison between the complex structure after 500 ns of simulation time (atomistic MD simulation, blue cartoon structures, three replicas) and the starting structure (shown as grey cartoon). The structures are fitted on their backbone atoms (Lower left panel: top view; lower right panel: side view).

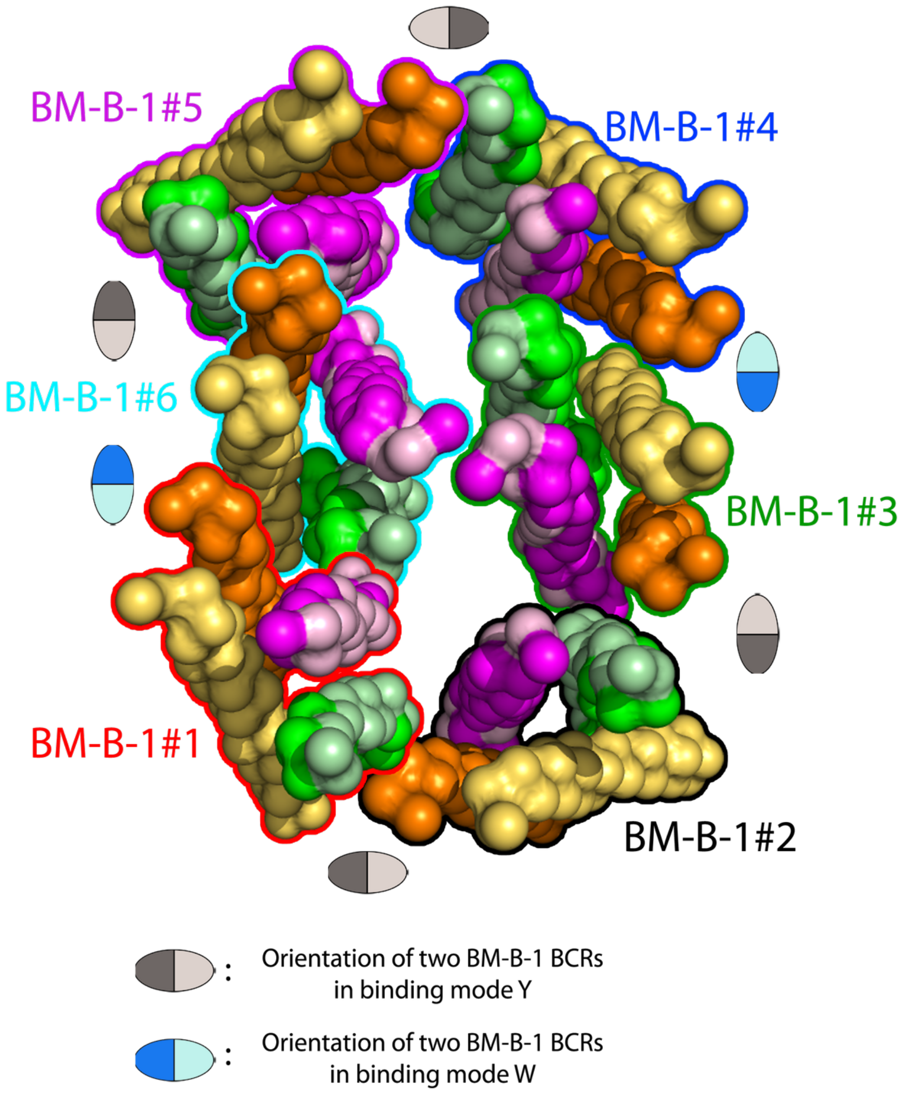

Figure S13. BCR TMD hexamer. Starting from a BCR dimer (BM-W conformer), the complex was extended stepwise by adding BCR TMDs in BM-Y and BM-W conformations. Following this procedure, complexes of arbitrary size can be modelled.
